# Alteration of Bacterial Wilt Resistance in Tomato Plant by Microbiota Transplant

**DOI:** 10.3389/fpls.2020.01186

**Published:** 2020-08-07

**Authors:** Kihyuck Choi, Jinhee Choi, Pyeong An Lee, Nazish Roy, Raees Khan, Hyoung Ju Lee, Hang Yeon Weon, Hyun Gi Kong, Seon-Woo Lee

**Affiliations:** ^1^ Department of Applied Bioscience, Dong-A University, Busan, South Korea; ^2^ School of Life Sciences, Forman Christian College (A Chartered University), Lahore, Pakistan; ^3^ Department of Biological Sciences, National University of Medical Sciences, Rawalpindi, Pakistan; ^4^ Agricultural Microbiology Division, National Institute of Agricultural Sciences, Rural Development Administration, Wanju, South Korea

**Keywords:** rhizosphere microbiome, tomato plant, microbiota transplant, bacterial wilt, *Ralstonia solanacearum*

## Abstract

Plant-associated microbiota plays an important role in plant disease resistance. Bacterial wilt resistance of tomato is a function of the quantitative trait of tomato plants; however, the mechanism underlying quantitative resistance is unexplored. In this study, we hypothesized that rhizosphere microbiota affects the resistance of tomato plants against soil-borne bacterial wilt caused by *Ralstonia solanacearum*. This hypothesis was tested using a tomato cultivar grown in a defined soil with various microbiota transplants. The bacterial wilt-resistant Hawaii 7996 tomato cultivar exhibited marked suppression and induction of disease severity after treatment with upland soil-derived and forest soil-derived microbiotas, respectively, whereas the transplants did not affect the disease severity in the susceptible tomato cultivar Moneymaker. The differential resistance of Hawaii 7996 to bacterial wilt was abolished by diluted or heat-killed microbiota transplantation. Microbial community analysis revealed the transplant-specific distinct community structure in the tomato rhizosphere and the significant enrichment of specific microbial operational taxonomic units (OTUs) in the rhizosphere of the upland soil microbiota-treated Hawaii 7996. These results suggest that the specific transplanted microbiota alters the bacterial wilt resistance in the resistant cultivar potentially through a priority effect.

## Introduction

The lethal bacterial wilt (BW) disease is caused by *Ralstonia solanacearum*, and the bacterial pathogen infects more than 400 plant species, especially plants belonging to the *Solanaceae* family (Hayward, 1991; Scott et al., 2005). *R. solanacearum* is a soil-borne pathogen that enters the plant through wounds or elongation zones and subsequently resides in the xylem vessels to block water transport (Vasse et al., 1995). The pathogenesis of BW, including bacterial invasion and pathogen colonization of the xylem, is regulated by a highly complex and sophisticated signaling mechanism (Hikichi et al., 2017). As there are no effective chemical agents for managing BW, the disease is generally managed by crop rotation and disease-resistant plants (Hanson et al., 1998; Wang et al., 2000). There are several BW-resistant cultivars of tomato (Wang et al., 1998), pepper (Du et al., 2016), and eggplant (Salgon et al., 2017). One of the well-known BW-resistant tomato cultivars is Hawaii 7996, which exerts the most stable resistance against *R. solanacearum* infection by several major and minor quantitative trait loci (QTL) (Thoquet et al., 1996; Wang et al., 1998). However, the quantitative resistance to BW is not completely understood, and the genes and functions of QTL have not been characterized in Hawaii 7996 and other major crops. It is known that the performance of quantitative resistance in Hawaii 7996 is frequently influenced by environmental conditions such as the pathogen strain, temperature, and soil conditions (Wang et al., 2013).

The plant rhizosphere is the dynamic and complex interface between the plant root and soil. The plant rhizosphere serves as a niche where the soil microbiota derives nutrition from the plant (Dennis et al., 2010; Berendsen et al., 2012). Recent studies have demonstrated that diverse microorganisms are associated with the plant in the rhizosphere (Mendes et al., 2013) and form plant-specific microbial communities (Hassani et al., 2018). Various factors in the rhizosphere affect the composition of the microbial community (Marschner et al., 1986; Dennis et al., 2010). Plant functions, such as growth, development, and stress tolerance, are influenced by the rhizosphere microbiota (Lau and Lennon, 2012; Panke-Buisse et al., 2015; Robbins et al., 2018). The soil microbiota in this niche can affect plant health negatively or positively. The agronomic goal is to positively promote plant functions including plant growth and health (Mendes et al., 2011; Lundberg et al., 2012; Bulgarelli et al., 2013). The plants shape the bacterial community structure of their rhizosphere using the microbial reservoir of the soil. Both biotic and abiotic factors are reported to shape the structural and functional diversities of microbial communities in the rhizosphere (Berg and Smalla, 2009; Bulgarelli et al., 2012; Lundberg et al., 2012).

Recently, soil microbiota was reported to protect plants against various diseases, such as potato scab disease caused by *Streptomyces* species (Meng et al., 2012), *Fusarium* wilt of various plants (Chialva et al., 2018), damping-off disease of sugar beet caused by *Rhizoctonia solani* (Mendes et al., 2011), and take-all decline of wheat caused by *Gaeumannomyces graminis* var. *tritici* (Weller et al., 1988). There is growing evidence that suggests the role of the rhizosphere microbiome in protecting the plant against soil-borne diseases (Kyselková et al., 2009; Chialva et al., 2018; Kwak et al., 2018). The soil microbiome can directly protect plants against disease or can modulate the plant’s defense mechanism against disease (Millet et al., 2010; Mendes et al., 2011). Additionally, plant defense hormones, such as salicylic acid, can modulate the soil microbial communities (Lebeis et al., 2015).

Soil is a highly heterogeneous matrix that supports plant growth. The physicochemical properties and microbial diversity of soil vary with each soil type. Although soil properties also contribute to plant growth and health, the physicochemical properties frequently mask the microbial function that regulates plant traits. Therefore, it is necessary to investigate the microbial function in the rhizosphere under defined soil conditions to understand the role of microbiota in regulating plant traits (Vorholt et al., 2017; Kwak et al., 2018).

In this study, we used soil microbiota transplant in tomato plants under defined soil condition to investigate the disease progress of BW in tomato. We have previously shown that microbial community structure of BW-resistant Hawaii 7996 is distinct from that of BW-susceptible cultivar Moneymaker, and specific microbiota is recruited by host plant to protect themselves (Kwak et al., 2018). However, in this study, we focused on the microbiota associated with the resistant cultivar Hawaii 7996 to investigate the role of tomato rhizosphere microbiota in influencing BW resistance. Our hypothesis is that soil microbiota transplant contributes to the formation of distinct rhizosphere microbial communities and to subsequently affect plant traits, especially BW resistance in tomato. To our knowledge, this is the first to show that plant quantitative trait can be affected by plant-associated microbiota.

## Materials and Methods

### Soil Sampling

In this study, we established a protocol to use soil microbial fraction (MF) for microbiota transplant from various soils. The initial soil samples comprised 18 different soils and included natural soils from different types of vegetation, such as various crop cultivated fields, forest, and alluvial soils from river estuarine and pasture areas, where there was no crop cultivation (**Supplementary Table S1**). The soil MFs were subjected to preliminary screening to determine the influence of soil microbiota on tomato BW progress. Based on these results, four different soils showing distinct and differential BW resistance by microbiota transplant were selected for further investigation: upland, paddy, forest, and alluvial soils. The topsoil (3–5 cm) and organic debris were removed, and the soil layer between 5 and 10 cm was collected using a shovel. The harvested soil was sieved through a 5 mm mesh to exclude the remaining organic debris. The sieved soil samples were stored in zipper bags at ambient temperature. For long-term storage, the soil samples were stored at −80°C in zipper bags under dark conditions until further use. The physicochemical properties of the soil samples were analyzed at the National Instrumentation Center for Environmental Management (NICEM), Seoul National University, Seoul, Korea. The physicochemical properties of each soil are listed in **Supplementary Table S2**.

### Preparation of the Soil MF

The soil MF was isolated from the soil samples using 170 g of soil. The soil sample was incubated with 250 ml of 2.5 mM MES buffer (pH 5.7) on a shaker at 200 rpm for 30 min. The mixture of soil and MES buffer was centrifuged at 500 rpm for 5 min to remove most of the soil particles. The supernatant was subsequently centrifuged again at 8,000 rpm for 15 min to collect the bacterial cell pellet. The bacterial cell pellet was resuspended in 220 ml of 2.5 mM MES buffer (pH 5.7) (**Supplementary Figure S1A**). This final bacterial suspension derived from 170 g of soil was used as the soil MF for treating 10 tomato seedlings and for the comparison of bacterial community between bulk soil and soil MF (**Supplementary Figure S2**).

### The Analysis System for Plant–Microbiome Interaction (ASPMI)

Tomato seeds (*Solanum lycopersicum* cv. Hawaii 7996 and cv. Moneymaker) were subjected to serial surface sterilization with 70% ethanol containing 0.1% TritonX-100 by vigorous vortexing for 1 min and 0.5% NaOCl containing 0.1% Triton X-100 for 15 min. The seeds were thoroughly washed with sterilized distilled water (SDW) and dried in a laminar flow hood before germination. The germination and plant growth conditions were 14 h/10 h of a light/dark regime at 28°C for all experiments. The seeds were germinated on sterilized filter paper in Petri dishes containing 5 mL SDW (ADVANTEC, Tokyo, Japan) for 7 days until planting. The germinated seedlings were planted in a 10 hole cell seedling tray that was surface-cleaned with 70% ethanol, and each hole contained 17 g of sterilized commercial horticultural nursery soil (Punong Co., Ltd, Korea). The horticultural nursery soils were autoclaved twice (121°C for 40 min) with an interval to allow the soil to reach ambient temperature before the second round of autoclaving. The planted tomato seedlings were treated with 20 ml of soil MF and were grown for 3 weeks before *R. solanacearum* inoculation. For the control, the seedlings were treated with an equal volume of 2.5 mM MES buffer (pH 5.7) (**Supplementary Figure S1B**). When necessary, the soil MF was diluted 10- or 100-fold with 2.5 mM MES buffer or was autoclaved at 121°C for 20 min before treatment to tomato seedlings.

### BW Disease Incidence Assays and Quantification of *R. solanacearum* SL341

All strains of *R. solanacearum* (**Supplementary Table S3**) were cultured in CPG medium plates containing 2,3,4-triphenyl tetrazolium chloride (TZC) for 36 h at 30°C. Except for the virulence comparison of Hawaii 7996 among *R. solanacearum* strains, the strain SL341 (race 1, phylotype I, *i.e.*, *R. pseudosolanacearum*) (Safni et al., 2014) was used for most BW progress assays (**Supplementary Table S3**). The cultured bacterial cells were suspended in SDW, and the cell density was adjusted to 2 × 10^8^ CFU/ml. The final bacterial suspension was poured onto the soil in the pot containing soil MF-treated plants (grown for 3 weeks after soil MF treatment) at a final concentration of 1 × 10^7^ CFU/g of soil (**Supplementary Figure S1B**).

To investigate the population of *R. solanacearum* SL341 in the tomato rhizosphere and endosphere, Hawaii 7996 grown in sterilized nursery soil were treated with upland soil MF or forest soil MF, and then after 3 weeks, SL341 strain was inoculated. SL341 cell density was measured at 2 h, 5 and 14 days post inoculation (dpi); the population of the cells was determine 2 h post inoculation in rhizosphere, and the cell density in the roots and stems of Hawaii 7996 cultivar was quantified at 5 and 14 dpi. BW disease incidence was scored until 14 dpi using the following formula: (number of wilted leaves/total number of leaves) × 100 (%). For disease scoring, three replications were used, each containing 10 plants for the soil MF treatment and control.

### Evaluation of the Antimicrobial Effect of the Rhizosphere Microbiome

In order to investigate whether the differential bacterial wilt resistance in Hawaii 7996 treated with soil MFs was due to direct antagonism to *R. solanacearum*, the antimicrobial activity of the rhizosphere soil of Hawaii 7996 treated with either upland soil MF or forest soil MF was tested. The soil MF-treated Hawaii 7996 cultivars were allowed to grow for 3 weeks. The rhizosphere soil was collected from the tomato plants. *R. solanacearum* SL341 at an OD_600_ of 0.3 was mixed with 32.5 ml of collected rhizosphere soil suspension in 2.5 mM MES buffer. This mixture was applied to 30 g sterilized nursery soil that was subsequently incubated at 30°C in a stationary incubator. The inoculated soil (1 g) from three replicates was collected to measure the colony forming units (CFUs) of *R. solanacearum* every 2.5 h until 10 h on semiselective SMSA medium (Engelbrecht, 1994).

### DNA Extraction From Bulk Soil, MF, and Rhizosphere Soil

Metagenomic DNA was extracted from 500 mg of bulk soil, plant rhizosphere and MF to amplify 16S rRNA genes. The tomato plants were manually harvested from the pots to collect the rhizosphere soil. The large soil aggregates loosely attached to the roots were removed by gentle tapping, leaving only the firmly adhered soil particles. The plant roots were immersed in 5 ml of 2.5 mM MES buffer (pH 5.7) in 50 ml falcon tubes and sonicated at 135 W for 5 min using a sonicator (Branson 5500DTH, Danbury, USA). Next, 5 ml of the soil suspension was centrifuged at 13,000 rpm. The soil pellet was weighed and processed for DNA extraction using the FastDNA™ SPIN for soil kit (MP Biomedicals, Solon, USA) following the manufacturer’s instructions.

### Amplicon Library Preparation and Sequencing

The 16S rRNA gene was subjected to GS-FLX amplicon sequencing and Illumina sequencing. Sequencing of samples (comparison of the bacterial community between bulk soil and MF in **Supplementary Figure S2**) was conducted as follows: For 454 pyrosequencing of the 16S rRNA gene amplicon, a PCR amplicon library was generated using the 341F (5′-TCGTCGGCAGCGTCAGATGTGTATAAGAGACAGCCTACGGGNGGCWGCAG-3′) and 805R (5′-GTCTCGTGGGCTCGGAGATGTGTATAAGAGACAGGACTACHVGGGTATCTAATCC-3′) (Mizrahi-Man et al., 2013) primers. These primers amplify a region spanning approximately 400 bp of the hypervariable region (V3–V4 region) of the bacterial 16S rRNA gene with the addition of 33 and 34 mer adaptors (underlined). Polymerase chain reaction (PCR) was performed in a 25-µl reaction volume containing 2.5 µl of 5 ng/µl template DNA, 12.5 µl of 2× KAPA HiFi HotStart Ready Mix (KAPA Biosystems), and 5 µl (1 µM) of each primer. The PCR conditions were as follows: initial denaturation at 95°C for 3 min, followed by 25 cycles of denaturation at 95°C for 30 s, annealing at 55°C for 30 s, and extension at 72°C for 30 s, with a final extension step at 72°C for 5 min.

Sequencing of the majority samples except for the data shown in **Figure S2** was conducted using Illumina (MiSeq) paired-end sequencing. For Illumina sequencing of the 16S rRNA gene amplicon, amplicon libraries were developed using the PCR primers 341F and 805R (Herlemann et al., 2011). These primers were used to amplify a region spanning approximately 400 bp of the hypervariable region (V3–V4 region). PCR was performed in a thermal cycler (Gene Atlas, Astec—Japan) in a 25-µl reaction volume containing 2.5 µl of 5 ng/µl template DNA, 12.5 µl of 2× KAPA HiFi HotStart Ready Mix (KAPA Biosystems), and 5 µl (1 µM) of each primer. The PCR conditions were as follows: an initial template denaturation step at 95°C for 3 min, followed by 25 cycles of denaturation at 95°C for 30 s, annealing at 55°C for 30 s, and extension at 72°C for 30 s, with a final extension step at 72°C for 5 min. To remove traces of PCR primers and primer dimers, PCR amplicons were purified using the Agencourt AMPure XP PCR Purification system (Beckman Coulter, Brea, USA), following the manufacturer’s instructions. The quality of amplicons (including the negative control) was evaluated by agarose gel electrophoresis using 1% gel. The DNA concentration was measured using a NanoDrop instrument (Thermo Scientific, Wilmington, MA). The libraries for paired-end sequencing and 454 pyrosequencing were prepared, and sequencing was performed at NICEM. The 16S rRNA gene amplicon sequences were analyzed for the microbial community structure as described in the supporting information. Microbiome network was analyzed using the Molecular Ecological Network Analysis (MENA) pipeline (Zhou et al., 2010) as described in the supporting information.

### Statistical Analysis

All statistical analyses were performed with R software (version 3.2.2) (http://www.r-project.org/). The suitability of the alpha-diversity indices was examined using the Shapiro–Wilk normality test followed by one-way univariate analysis of variance (ANOVA) and Tukey’s honestly significant difference (HSD) *post hoc* test in R. As the data were not normally distributed, statistically significant differences in alpha-diversity indices were examined by nonparametric Kruskal–Wallis one-way ANOVA followed by Dunn’s multiple comparisons *post hoc* test. To identify taxa that were significantly different between the Hawaii 7996 rhizosphere microbiota under the ASPMI treated with two different soil MFs, we used DESeq2 package. DESeq2 was run under a negative binomial fit, and Wald test and q-values were calculated with the Benjamini–Hochberg procedure to correct *p*-values and control for false discovery rates. Differences in the abundance were considered significant when FDR adjusted *p*-values were lower than 0.0001. The significant differences among the four different bulk soil and MF treatment groups (alluvial, forest, paddy, and upland) were evaluated by multivariate analysis of variance using distance matrices (ADONIS), which calculates squared deviations and determines statistical significance by F-tests on sequential sums of squares from permutations of data.

## Results

### Establishment of ASPMI

Soil MFs from the various natural bulk soils were collected from several places and added to the sterilized soil to grow tomato seedlings with the isolated soil MFs (**Supplementary Figures S1A, B**). This ASPMI method enables the investigation of plant traits relevant to the treated microbiota in sterilized soils with similar physicochemical properties containing various microbiota. To verify the utility of ASPMI, thirteen physiochemical properties were comparatively evaluated between the four different field soils and sterilized commercial nursery soils treated with the MFs isolated from the corresponding four field soils. The four field soils exhibited differential physicochemical properties. However, the physicochemical properties were similar between the sterilized nursery soils that were treated with the four soil MFs (**Supplementary Table S2** and **Supplementary Figure S3**). These results suggest that the ASPMI method successfully eliminated the differential effect of physicochemical properties among the field soil samples, which enabled the evaluation of plant–microbiota interactions under controlled soil conditions using various soil MFs.

The bacterial community structure of both soil MFs and bulk soils was compared to determine whether the isolated soil MF represented the microbiota of the respective bulk soil. Principal coordinate analysis (PCoA) based on the Bray–Curtis dissimilarity measure was performed to determine the beta-diversity (community comparison among microbial community) of the microbiome in the bulk soils and the corresponding MFs. The PCoA revealed that each resultant microbiota separated across the first and second principal coordinates (22.6 and 17.8% of variation, respectively), whereas only limited separation was observed between bulk soil and its respective MF (**Supplementary Figure S2**). Therefore, this indicated that the isolated soil MFs represented the bacterial community of the respective bulk soils.

### Microbiota Transplant Influences BW Resistance

**Figure 1:**
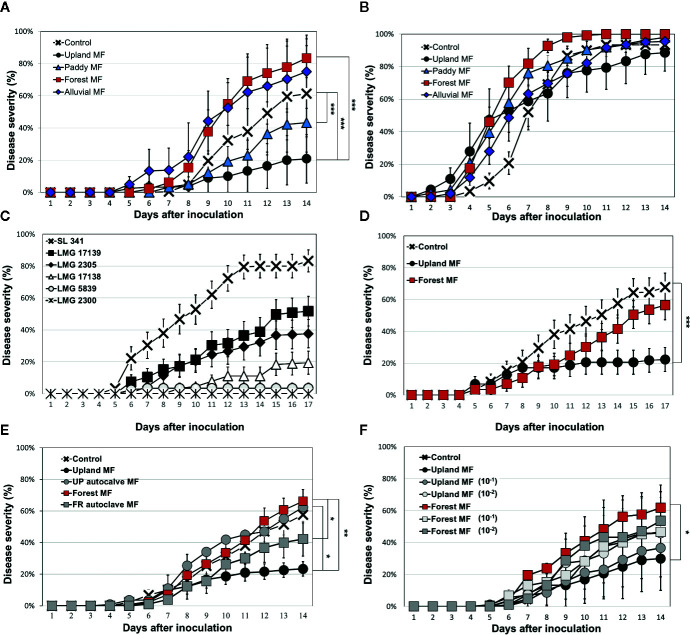
Bacterial wilt (BW) disease progression in tomato cultivars treated with soil microbial fractions (MFs) evaluated by ASPMI method. BW disease progression by *R. solanacearum* SL341 in the tomato cultivar Hawaii 7996 treated with four different soil MFs **(A)** and in the susceptible cultivar Moneymaker treated with soils MFs **(B)**. BW disease progression in the Hawaii 7996 cultivar inoculated with six different *R. solanacearum* strains **(C)**. Evaluation of BW disease progression by *R. solanacearum* LMG 17139 strain in Hawaii 7996 treated with upland soil MF or forest soil MF **(D)**. Effect of heat-killed soil MFs (autoclave MF) **(E)** on BW disease progression in the Hawaii 7996 cultivar. Effect of diluted soil MF **(F)** on BW disease progression in the Hawaii 7996 cultivar (10^−1^, ten-fold diluted MF, 10^−2^, hundred-fold diluted MF). Control was treated with 2.5 mM MES buffer (pH 5.7). Each data point represents the mean disease incidence from three independent experiments. In total, 30 plants were analyzed for each treatment. Each vertical bar represents the standard error of the mean from three replicates (each replicate with 10 plants, *n* = 30). Significant difference was evaluated by repeated measure analysis of variance (ANOVA) (*p < 0.05; **p < 0.01; ***p < 0.001).

The ASPMI method was used to test the differential effect of various soil MFs against BW disease in the BW-resistant tomato cultivar (Hawaii 7996) and BW-susceptible cultivar (Moneymaker). In this study, 18 different soil MFs from various natural ecosystems in Korea were used to test BW resistance by inoculation of *R. solanacearum* strain SL341. Each microbiota transplant exhibited differential BW progress in the resistant cultivar Hawaii 7996 (**Figure 1A** and **Supplementary Figure S4**). To further investigate the effect of microbiota on BW resistance on Hawaii 7996, four different soils from various natural ecosystems in Korea were selected based on the distinct and differential quantitative resistance of tomato BW and the reproducibility of the results after several repetitive experiments. The BW disease progression was significantly different among the soil MF-treated Hawaii 7966 plants. Interestingly, the progression of BW in the upland soil MF and paddy soil MF-treated plants was significantly delayed (repeated measures ANOVA, *p* < 0.001) compared to that in the control (**Figure 1A**). Treatment of upland soil MF was more effective to suppress disease progress in the Hawaii 7996 cultivar The forest soil MF-treated Hawaii 7996 plants exhibited the highest susceptibility to BW disease. However, the progression of BW in Moneymaker plants was similar among the plants treated with different soil MFs (**Figure 1B**).

**Figure 2:**
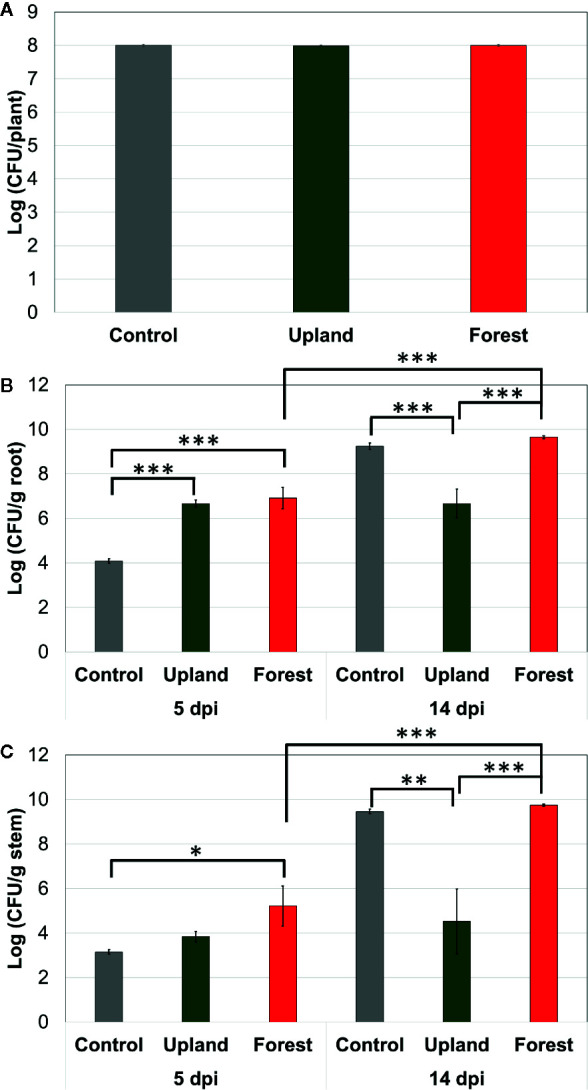
Population dynamics of *R. solanacearum* SL341 in the tomato rhizosphere **(A)**, roots **(B)**, stems **(C)** of resistant cultivar treated by upland soil MF and forest soil MF over time. Control was treated with 2.5 mM MES buffer (pH 5.7). Each vertical bar represents the standard error of the mean from three replicates (each replicate with three plants, n = 3) (*p < 0.05; **p < 0.01; ***p < 0.001).

Bacterial population of SL341 was estimated at three different time points in rhizosphere and endosphere of tomato plant (*cv*. Hawaii 7996). Initial inoculum densities of SL341 at 2 h post inoculation were not significantly different among treatments (**Figure 2A**), which is equivalent to 10^7^ CFU/g of soil. Bacterial population of SL341 in the tomato roots was not significantly different at 5 dpi between upland soil MF transplant and forest soil MF transplant, while that with forest soil MF transplant was significantly higher than that with upland soil MF transplant at 14 dpi (**Figure 2B**). The population of SL341 was significantly increased by forest soil MF transplant from 5 dpi to 14 dpi. However, the population of SL341 was maintained steady from 5 dpi to 14 dpi by upland soil MF transplant (**Figure 2B**). Similarly, bacterial population of SL341 in the tomato stems was not significantly different at 5 dpi between upland soil MF transplant and forest soil MF transplant. The bacterial population was significantly increased from 5 dpi to 14 dpi by forest soil MF transplant whereas that transplanted by upland soil MF was maintained steady (**Figure 2C**). Overall, the bacterial population of SL341 in tomato roots and stems treated with different soil MFs was coincident to the bacterial wilt progress in Hawaii 7996 (**Figure 1A**).

Hawaii 7996 is highly resistant to *R. solanacearum* strains; however, this cultivar was susceptible to strain SL341 in sterile soil without microbiota transplantation. Therefore, we tested whether other strains of *R. solanacearum* could affect BW resistance under the same conditions. Six strains of *R. solanacearum* were selected for the virulence test (**Supplementary Table S3**) based on the stable production of exopolysaccharide (EPS) in TZC medium. Among these six strains, the LMG17139 strain exhibited the highest virulence except SL341 strain in the Hawaii 7996 cultivar (**Figure 1C**). Furthermore, the upland soil MF-treated Hawaii 7996 cultivar inoculated with the LMG17139 strain exhibited significantly (repeated measures ANOVA, *p* < 0.001) delayed BW disease progression compared to the control and forest soil MF (**Figure 1D**). No significant difference of disease progress was observed between control and forest soil MF. Although it is speculative, the vulnerability of BW resistance in Hawaii 7996 to highly virulent strains of *R. solanacearum* could be altered by soil microbiota transplant.

Next, the effect of soil MF on BW disease progression was evaluated by treating the Hawaii 7996 cultivar with autoclaved (heat-killed) soil MFs. The heat-killed soil MFs completely diminished the suppression or induction of disease progression observed in the plants treated with live soil MFs (**Figure 1E**). No significant difference was noticed among treatments of heat-killed soil MFs. Moreover, the treatment with diluted (10^−1^ and 10^−2^) soil MF decreased the positive or negative effect on the BW disease progression and equalized the disease severity (**Figure 1F**). Although each soil MF affected BW disease progression in the BW-resistant Hawaii 7996 cultivar but not in the BW-susceptible cultivar, we needed to test whether the rhizosphere microbiota of Hawaii 7996 treated by upland soil MF would inhibit the growth of bacterial pathogens by direct antagonistic effects. To test this, we incubated *R. solanacearum* SL341 in the rhizosphere soils from Hawaii 7996 cultivars treated with an upland soil MF or a forest soil MF. However, direct growth inhibition of *R. solanacearum* was not observed in the rhizosphere soils (**Supplementary Figure S5**).

### Effect of Transplanted Soil MFs on the Microbiota Structure in the Tomato Rhizosphere

**Figure 3:**
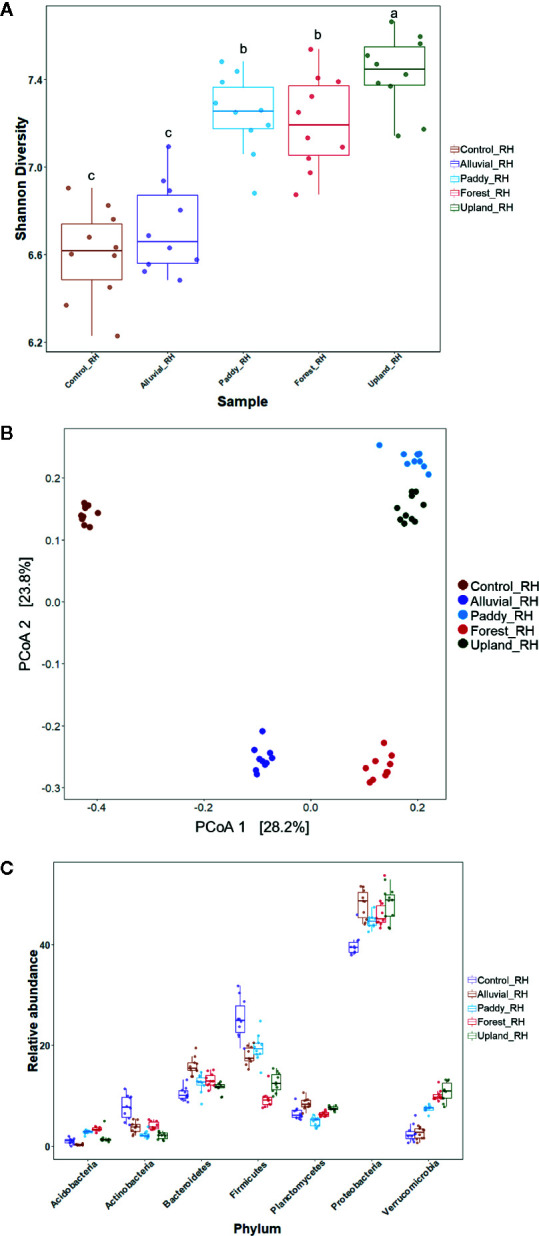
Microbial community analysis of rhizosphere in the Hawaii 7996 cultivar treated with different soil microbial fractions (MFs). **(A)** Comparative alpha-diversity analysis of bacterial community among the bacterial wilt (BW)-resistant Hawaii 7996 cultivars treated with four different MFs. Shannon diversity index (*H′*) is calculated after subsampling (*p* < 0.05, ANOVA with HSD *post hoc* test). Different letters indicate significant differences. **(B)** Comparative beta diversity analysis of bacterial community among the BW-resistant Hawaii 7996 cultivars treated with four different MFs. The distribution pattern of four rhizosphere microbiomes in the BW-resistant cultivars evaluated using nonmetric multidimensional scaling based on the Bray–Curtis dissimilarity measures. **(C)** Relative abundance (%) of the major bacterial phyla in the rhizosphere microbiota of the BW-resistant cultivar treated with four different soil MFs.

The effect of soil MF or MES buffer treatment (control) on the bacterial community structure was investigated in the tomato rhizosphere. Comparative analysis of alpha-diversity indices [Shannon diversity index (*H′*)] revealed that there was a significant difference in the alpha diversity of the tomato rhizosphere microbiota (ANOVA with HSD *post hoc* test, *p* < 0.05), except between the rhizosphere microbiota of control and alluvial soil MF-treated plants and between the rhizosphere microbiota of paddy soil MF-treated and forest soil MF-treated plants. The alpha diversity of the rhizosphere microbiota of control plants exhibited the lowest *H′*, whereas that of upland soil MF-treated plants exhibited the highest *H′* (**Figure 3A**). Bray–Curtis dissimilarity multivariate analysis was performed for tomato rhizosphere microbiota post soil MF treatment. Nonmetric multidimensional scaling (NMDS) was used to visualize the microbial community structure. Distinct separation of bacterial communities in the rhizosphere was apparent in the tomato plants treated with different soil MFs (**Figure 3B**). ADONIS revealed that the rhizosphere microbiota of Hawaii 7996 exhibited significant differences in the microbial community structure between the groups (R^2^ = 0.37946, *p* < 0.001).

Additionally, the rhizosphere microbiota of tomato plants was distinct from the microbiota of bulk soils in the same pot, which contained the initially treated MFs. There was no significant difference in species richness and evenness between the rhizosphere and bulk soils treated with upland soil MF and forest soil MF (**Supplementary Figure S6A**). Bray–Curtis dissimilarity measures revealed that there was a significant difference (R^2^ = 0.37838, *p* < 0.001) in the microbial communities among bulk soils and rhizosphere soils of tomato plants treated with the upland soil MF or forest soil MF (**Supplementary Figure S6B**).

These results suggest that the rhizosphere microbiota in the tomato plants was sculpted to have a unique community structure from the respective soil MF input with a distinct community composition.

### Microbial Taxa Distribution in the Rhizosphere of Soil MF-Treated Tomato

The microbial community composition of the tomato rhizosphere was comparatively evaluated between the upland soil MF-treated and forest soil MF-treated plants. The rhizosphere microbiota of the upland soil MF-treated tomato plants was different from that of forest soil MF-treated tomato plants (**Figures 3A, B**). The rhizosphere of upland soil MF-treated and forest soil MF-treated tomato plants exhibited significant differences in the relative abundance (RA) of the following bacterial phyla: *Acidobacteria* (1.60 and 3.34%, respectively), *Actinomycetes* (2.05 and 4.00%, respectively), *Planctomycetes* (7.49 and 6.33%, respectively), *Firmicutes* (12.6 and 9.32%, respectively) (**Figure 3C**). Further, individual taxa displaying differential abundance are listed in **Supplementary Table S4** (DESeq2, log2 fold change); plus log2 fold change indicates the individual taxa belongs to *Opitutaceae*, *Burkholderiaceae*, *Oxalobacteriaceae*, *Pseudomonadaceae*, *Xanthomonadaceae*, *Chitinophagaceae*, and *Planctomycetes* which are more abundant in upland soil MF-treated tomato plants. The minus log2 fold change indicates the individual taxa belongs to *Enterobacteriaceae*, *Calulobacteriaceae*, *Chthoniobacteriaceae*, *Chitinophagaceae*, *Isosphaeraceae*, *Cytophagaceae, and* *Sporolactobacillaceae* which are more abundant in forest soil MF-treated tomato plants (**Supplementary Table S4**). Particularly, 118 OTUs were enriched in upland soil MF-treated tomato plants, and 88 OTUs were enriched in forest soil MF-treated tomato plants (**Supplementary Table S4**). These data illustrate that certain rhizosphere bacterial OTUs or a combination of OTUs may be responsible for the differential disease progress of BW in Hawaii 7996.

### Putative Keystone Taxa and Their Differential Abundance in the Tomato Rhizosphere

**Figure 4:**
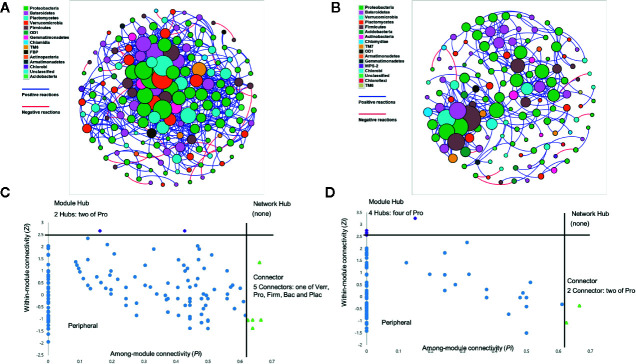
Co-occurrence network of rhizosphere of tomato treated with upland soil microbial fraction (MF) **(A)** and forest soil MF **(B)**. Each node represents different operational taxonomic units (OTUs), while the edges indicate correlation between the nodes. Edges (lines) between nodes are colored blue for positive correlations between taxa; negative correlations are colored red. Node size corresponds to the number of edges. Node color represents each Phylum. Analysis of nodes to identify the putative keystone species in the rhizosphere networks **(C, D)**. Each symbol represents an OTU from rhizosphere network of upland soil MF-treated plants **(C)** and forest soil MF-treated plants **(D)** adopted for detailed module analysis. Network hub contains *Zi* > 2.5 and *Pi* > 0.62. Module hubs have *Zi* > 2.5, while the connectors retain *Pi* > 0.62. The taxonomy information of the module hubs (purple) and module connectors (green) is named on the plot using the following abbreviations; Bac, *Bacteroidetes*; Firm, *Firmicutes*; Plac, *Planctomycetes*; Pro, *Proteobacteria*; Verr, *Verrucomicrobia*.

The potential candidate OTUs for the network hub, module hub, and connector were identified based on the rhizosphere microbial community data of upland soil MF-treated (**Figure 4A**) and forest soil MF-treated tomato plants (**Figure 4B**). The module connectivity (*Zi*) and among-module connectivity (*Pi*) values (Deng et al., 2012) were measured in the rhizosphere soil of upland soil MF-treated and forest soil MF-treated Hawaii 7996 plants. The network analysis revealed that the peripherals were the most abundant nodes in the network hub. Additionally, no network hub was detected in the rhizosphere of upland and forest soil MF-treated tomato plants (**Figures 4C, D**). In total, two module hubs, OTUs of *Bacteroidetes*, were identified in the rhizosphere network of upland soil MF-treated plants. Five module connectors, OTUs of *Proteobacteria*, *Verrucomicrobia*, *Firmicutes*, *Bacteroidetes*, and *Planctomycetes* *Bacteroidetes* were also identified in the rhizosphere network of upland soil MF-treated Hawaii 7996 (**Figure 4C** and **Supplementary Table S5**). On the other hand, four module hubs belonging to *Proteobacteria* and two connectors, OTUs of *Proteobacteria* were detected in the rhizosphere network of forest soil MF-treated Hawaii 7996 (**Figure 4D** and **Supplementary Table S5**). The putative keystone taxa of the rhizosphere of upland soil MF-treated and forest soil MF-treated plants include OTUs from *Verrucomicrobia, Firmicutes, Bacteroidetes*, and *Planctomycetes* which were identified based on the *Pi* and *Zi* scores. However, they did not overlap in the network analysis between communities of two different treatment groups.

**Figure 5:**
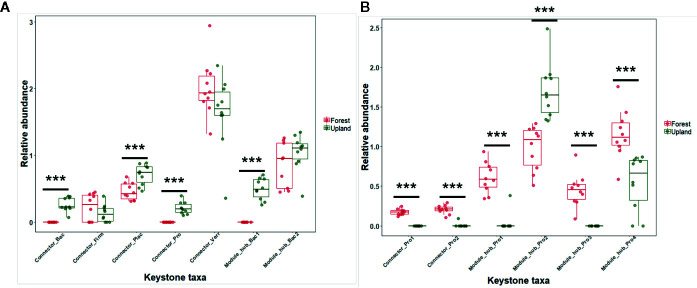
Relative abundance (%) of the keystone taxa in co-occurrence network of rhizosphere in the upland soil MF-treated plants **(A)** and forest soil MF-treated plants **(B)**. Statistical significances between the treatment groups were evaluated by Wilcoxon signed ranks test using the R software (version 3.2.2). ***p < 0.001.

The RA of microbes in the module hubs ranged from 0.496 to 1.042% for upland soil MF-treated plants and from 0.451 to 1.158% for forest soil MF-treated plants (**Supplementary Figure S7**). The connectors also exhibited low RA (0.131 and 1.641%) (**Supplementary Figure S7**). The RA of putative keystone taxa in each treatment group revealed the differential abundance of keystone taxa in the rhizosphere of upland soil MF-treated and forest soil MF-treated plants (**Figure 5**). The number of network topological properties was compared among the rhizospheres of tomato treated with four different soil MFs. The properties included total nodes, total edges, the average degree, network diameter, network density, average clustering coefficient, and average path length (**Supplementary Table S6**). The microbiota network of the rhizosphere of upland soil MF-treated plants exhibited a higher number of network topological properties than the rhizosphere of forest soil MF-treated plants (**Supplementary Table S6**).

## Discussion

This study aimed to test our hypothesis that resistance of the well-known tomato cultivar Hawaii 7996 against BW is altered by rhizosphere microbiota. Rhizosphere microbiota is highly complicated and dependent on the surrounding soils. The structure of the rhizosphere microbiota is influenced by various biotic and abiotic factors. The critical factors determining the structure of rhizosphere microbiota are the soil type and soil physicochemical properties (Schutter et al., 2001; Bulluck et al., 2002; Marschner et al., 2004; İnceoğlu et al., 2012). Because the physiochemical properties of soil strongly influence the rhizosphere microbiome structure (Staley and Konopka, 1985; Amann et al., 1995; Hugenholtz et al., 1998; Lauber et al., 2008), we established the ASPMI method to evaluate the BW resistance of tomatoes treated with various soil MFs under sterile soil conditions. A previous study isolated microbes from field soils by excluding variations in soil physicochemical properties and treated isolated microbes with *Boechera stricta* seedlings to determine their effect on flowering phenology and time (Wagner et al., 2014). Our study adopted and modified the method developed by Wagner et al. (2014). The modifications mostly included two-step centrifugation of the soil suspension instead of filtration of soil suspension to establish the ASPMI protocol (**Supplementary Figure S1**). Our ASPMI protocol successfully excluded the abiotic factors in various soils (**Supplementary Table S2**). It is likely that application of soil MFs to aseptically germinated tomato seedlings aided in microbial colonization of the rhizosphere of tomato to sculpt a unique rhizosphere microbial community in the sterilized soil. Therefore, the microbial community may exhibit a priority effect in the tomato rhizosphere (Busby et al., 2017; Wei et al., 2019) as the soil MF was applied to germ-free tomato seedlings.

The rhizosphere microbiota plays an important role in protecting plants from pathogen invasion (Kwak et al., 2018) by preventing pathogen colonization or by facilitating the colonization of commensal bacteria. In this study, the upland soil MF-treated Hawaii 7996 cultivars exhibited higher BW disease resistance than the plants treated with other soil MFs. Interestingly, the soil MF treatment did not protect the BW-susceptible cultivar Moneymaker (**Figures 1A, B**). However, the MFs of upland soil and forest soil did not exhibit any antimicrobial effect against *R. solanacearum* (**Supplementary Figure S5**). This suggested that the observed BW resistance in Hawaii 7996 was the function of plant–microbiota interaction and was not due to the direct antagonistic effect. In fact, population dynamics of *R. solanacearum* SL341 showed that population of SL341 was not different at 5 dpi both in the roots and stems of Hawaii 7996 treated with either upland soil MF or forest soil MF (**Figure 2**). This suggested that pathogen invasion was not affected by microbiota transplant in tomato roots, and there might be no antagonistic effect by upland soil MF to bacterial pathogen.

Interestingly, in the same BW-resistant tomato cultivar, bacterial population of SL341 *in planta* was dramatically increased over time by forest soil MF treatment but not by upland soil MF treatment (**Figure 2**). It is likely that microbiota transplant somehow influenced the BW resistance of tomato Hawaii 7996 to have altered disease progress, *i.e.* tomato plants with upland soil-derived microbiota hindered the multiplication of bacterial pathogen *in planta.* Several reports have illustrated that *R. solanacearum* in the resistant tomato cultivar is limited to colonize inside of tomato plants and not able to multiply in the stem, although the resistant cultivar contains significant number of bacteria in the roots and shoots (Grimault et al., 1994; Saile et al., 1997). The colonization of *R. solanacearum* race 3 in tomato stems restricted by QTL was also reported by Carmeille et al. (2006). Our result suggests that the priming of the defense response or alteration r of disease resistance trait is mediated by the rhizosphere microbiome in the Hawaii 7996 cultivar. The upland soil MF treatment conferred higher resistance to BW only in the BW-resistant cultivar, which further indicated that the alteration of BW disease resistance is mediated by the rhizosphere microbiome. One can argue if the differential entophytic community derived from different soil MFs transplant may be responsible for the altered BW-resistance. This needs to be investigated further. In contrast, the forest soil MF-treated Hawaii 7996 cultivars exhibited enhanced susceptibility to BW. It would be interesting to evaluate whether certain groups of microbial taxa or the microbial community enhance BW disease susceptibility in BW-resistant cultivars.

In this study, the ASPMI method was used to harvest microbes to obtain the soil MF from a variety of natural bulk soils and to analyze the biological effect of the isolated soil MF. In fact, the BW resistance of Hawaii 7996 conferred by upland soil MF transplant was completely abolished by heat-killed MF treatment. This result suggested that the plant phenotype (*i.e.*, BW resistance) could be regulated by the biological effect of treated soil MF. The tomato rhizosphere microbiota shaped by treating the sterilized nursery soil with various soil MFs may not fully represent the function of the original field soil microbiota because the soil properties under ASPMI were completely different from those of the original soil. However, the soil MF enabled the reproducible investigation of plant host response, *i.e.*, tomato BW resistance, to its microbiota compared to the field soil microbiota. This is because the field soil microbial composition is affected by fluctuating environmental factors that cannot be efficiently controlled. In this study, two soil MFs, upland soil MF and forest soil MF, displayed differential effects on the BW resistance of Hawaii 7996 under ASPMI. The bacterial community diversity and composition were markedly different between the fertilized, intensely managed grassland, and forest soils (Peiffer et al., 2013). Similarly, the rhizosphere bacterial community structure was significantly different between the upland soil MF-treated and forest soil MF-treated tomato plants (**Figure 3A**). These results indicate that the differential composition of bacterial taxa observed in the rhizosphere of the plants treated with various soil MFs may potentially influence the BW resistance. This differential effect on the plant phenotype may be due to the priority effect of initially colonized microbiota in the tomato rhizosphere (Wei et al., 2019).

In this study, most of the network nodes and connectors in the rhizosphere of upland soil MF-treated and forest soil MF-treated plants exhibited a relatively low abundance of putative keystone taxa (**Supplementary Figure S7**). This indicated that the rare taxa may be key to developing or maintaining the structure of the rhizosphere network. In fact, treatment of diluted upland soil MF lost the original upland soil MF activity of BW resistance (**Figure 1F**), suggesting that key players of upland soil MF to alter BW resistance in Hawaii 7996 could be members with low abundance. Similar conclusions were reported in other studies on microbial community analysis with macro- and micro-ecological networks in various ecosystems (Power et al., 1996; Lyons and Schwartz, 2001; Pester et al., 2010; Lupatini et al., 2014; Deng et al., 2016). The rhizosphere microbiome of upland soil MF-treated plants exhibited a higher complexity in the network in this study. An earlier study reported that the microbial community in the *Fusarium oxysporum* (*Fox*)-resistant cultivar was more complex than that in the *Fox*-susceptible cultivar (Mendes et al., 2017). High bacterial diversity was reported to confer enhanced resistance against pathogen invasion (Latz et al., 2012; Mallon et al., 2015). The disease resistance driven by the relationship between microbial diversity and pathogen invasion could be described by the fundamental interaction network architecture (Wei et al., 2015). The rhizosphere network of upland soil MF-treated plants contained more nodes and edges when compared to the rhizosphere network of forest soil MF-treated plants. The results of this study suggested that the highly connected and modular rhizosphere microbial community may be involved in conferring enhanced BW disease resistance to plants.

Genes and mechanisms underlying the quantitative resistance to BW in tomato Hawaii 7996 cultivar have yet to be identified and characterized. However, two major QTLs, *Bwr-12* and *Bwr-6*, have been identified in Hawaii 7996. *Bwr-12* confers phylotype I-specific BW resistance, and *Bwr-6* confers broad-spectrum BW resistance (Grimault and Prior, 1993). The *R. solanacearum* strain SL341 used in this study is phylotype I, and it is not clear how this strain could affect BW resistance in Hawaii 7996. Nonetheless, specific microbiota transplant, *i.e.*, upland soil MF, conferred stable resistance in Hawaii 7996 against the strain SL341. This result revealed the alteration of quantitative resistance in Hawaii 7996 by soil microbiota transplant, which will sculpt unique root microbiota. It would be interesting to investigate how the rhizosphere microbiota influences BW resistance in Hawaii 7996 once the genes are cloned. In conclusion, our ASPMI method can successfully be used to evaluate the effects of microbiota transplantation on the BW resistance of tomatoes, and this study is the first to show that quantitative traits of plant, such as disease resistance, can be altered by soil microbiota transplantation.

## Data Availability Statement

The datasets presented in this study can be found in online repositories. The names of the repository/repositories and accession number(s) can be found below: https://www.ncbi.nlm.nih.gov/sra/?term=prjna559787.

## Author Contributions

S-WL conceived, organized, and supervised the project. KC, JC, RK, PL, and S-WL interpreted the result and prepared the manuscript. KC, JC, PL, NR, RK, HL, and HK performed the experiments. KC, HW, HK, PL, and S-WL edited the manuscript. All authors contributed to the article and approved the submitted version.

## Conflict of Interest

The authors declare that the research was conducted in the absence of any commercial or financial relationships that could be construed as a potential conflict of interest.
