# Supplementary material for: Alteration of Bacterial Wilt Resistance in Tomato Plant by Microbiota Transplant
Source: Front Plant Sci. 2020 Aug 7;11:1186. doi: 10.3389/fpls.2020.01186 (PMC7427413; doi:10.3389/fpls.2020.01186)
Supplement: Supplementary file 2 [file DataSheet_2.docx]

Supplementary material for Modulation of quantitative resistance to tomato bacterial wilt by microbiota transplant

**Kihyuck Choi^1Ϯ^, Jinhee Choi^1Ϯ^, Pyeong An Lee^1^, Nazish Roy^1,2^, Raees Khan^1,3^, Hyoung Ju Lee^1^, Hang Yeon Weon^4^, Hyun Gi Kong^4^, and Seon-Woo Lee^1^***

*^1^Department of Applied Bioscience, Dong-A University, Busan 604-714, Republic of Korea*

*^2^School of Life Sciences, Forman Christian College (A Chartered University), Lahore 54600, Pakistan*

*^3^Department of Biological sciences, National University of Medical Sciences, Rawalpindi 46000, Pakistan*

*^4^Agricultural Microbiology Division, National Institute of Agricultural Science, Rural Development Administration, Jeollabuk-do 55365, Republic of Korea*

^Ϯ^These authors contributed equally to this work.

*** Correspondence:**

* Corresponding author

Seon-Woo Lee, Ph.D

Professor

Department of Applied Bioscience

Dong-A University

Busan 49315

Republic of Korea

Email: seonlee@dau.ac.kr

**Supplementary materials and methods**

*Sequence analysis using DADA2, VEARCH, and QIIME2*

DNA sequences generated by 454 for the generating supplementary Fig. 2 and Illumina (all Figures and tables except supplementary Fig. 2) were de-multiplexed by NICEM. To remove low quality of sequences, filter chimeric sequences and merge the filterd-forward and reverse sequences, dada2 (dada2 denoise-paired) plug in the the QIIME2 (version 2018.06) pipeline was employed; filtering parameters were maxN=0, truncQ=2, rm.phix=TRUE, maxEE=2 and truncLen (280, 240) for the sequences for Hawaii 7996 rhizosphere treated by upland MF, paddy MF, forest MF and alluvial MF (Callahan et al., 2016). After the quality filtering, removing chimera, and merging steps completes, the high-quality merged sequences were clustered into operational taxonomy units (OTUs) using the de nove VSEARCH algorithm (vsearch cluster-features-de-novo) (Rognes et al., 2016) using a threshold of 97% pair-wise nucleotide sequence identity and then assembled into an OUT table for the further downstream analysis in R. The taxonomy of the non-chimeric OTUs was assigned using Naïve Bayes algorithm implemented in the q2-feature-classifier prefitted to the Greengens database (gg_13_5) for V3-V4 region of 16S rRNA regions (DeSantis et al., 2006).

*Alpha-diversity, Beta-diversity, and Relative abundance analysis*

The rarefy_even_depth function of the Phyloseq package using OTU table as input file was used to make an even depth of reads after subsampling. Bacterial alpha-diversity (Shannon index (*H’*)) was calculated from the rarefied OTU table using the estimate_richness function of the Phyloseq (McMurdie et al., 2013). Beta-diversity (Bray-Curtis dissimilarity measures) was calculated with a normalized OTU table employing CSS method with QIIME script CSS function normalize_table.py. Relative abundance analysis was performed with normalized OTUs. Individual OTUs in each sample were normalized by dividing the total counts of all OTUs within that sample, indicating relative abundance (RA) expressed as the taxonomy composition of each sample.

*Network analysis*

Network analysis was performed to compare each MF treatment based on OTUs relative abundances. Covariations were measured across 10 biological replications to construct each network. Only OTUs detected in 6 out of 10 replicates samples were employed for network construction. Random Matrix theory (RMT) was used to identify the appropriate similarity threshold (*St*) defining the minimal strength of the connections between each pair of nodes prior to network construction (Deng et al., 2012). All network analysis were performed using the Molecular Ecological Network Analyses (MENA) Pipeline (<http://ieg2.ou.edu/MENA/>) (Zhou et al., 2010) and visualization of the network analysis output was performed using Cytoscape 3.4.0 (Shannon et al., 2003) and Gephi 0.9.1-beta (https://github.com/gephi).

*Identification of network hubs, module hubs and connectors*

We identified network and module hubs and connectors in each network analysis. A module is a group of nodes (OTUs) which possesses numerous connections within the group and is rarely connected outside the group (Peiffer et al., 2013). In this study, the modules were identified using the greedy modularity optimization method (Deng et al., 2012). Modularity (*M*) was employed to analyze the extent to which a network is separated into modules as an index, we used M > 0.4 as the threshold to define into modular structures (Newman et al., 2006). The modular properties of each node in the network analysis were assigned by within-module connectivity (*Zi*) and among-module connectivity (*Pi*). These properties were employed to classify the topological roles of nodes in the network (Deng et al., 2012; Lundberg et al., 2012). The node role was classified into four categories: Peripheral nodes (*Zi* < 2.5 and Pi < 0.62), which display few outside connections; Connectors (*Zi* < 2.5 and *Pi* > 0.62), which are highly connected to nodes; module hubs (*Zi* > 2.5 and *Pi* < 0.62), which are highly liked to nodes within module; network hubs (*Zi* > 2.5, *Pi* > 0.62), which are highly connected to nodes in all network (Zhou et al., 2010; Deng et al., 2012; Wagner et al., 2016).

**Supplementary material References**

Callahan, BJ., McMurdie, PJ., Rosen, MJ., Han, AW., Johnson, AJA., Holmes, SP. (2016). DADA2: high-resolution sample inference from Illumina amplicon data. *Nat Methods*. 13, 581.

Caporaso, JG., Kuczynski, J., Stombaugh, J., Bittinger, K., Bushman, FD., Costello, EK., Fierer, N., Pena, AG., Goodrich, JK., Gordon, JI., Huttley, GA. (2010). QIIME allows analysis of high-throughput community sequencing data. *Nat. Methods*. 7, 335-336.

Deng, Y., Jiang, Y-H., Yang, Y., He, Z., Luo, F., Zhou, J. (2012). Molecular ecological network analyses. *BMC Bioinformatics* 13, 113.

DeSantis, Todd Z., et al. (2006) Greengenes, a chimera-checked 16S rRNA gene database and workbench compatible with ARB. *Appl. Environ. Microbiol*. 72, 5069-5072.

Lundberg, DS., Lebeis, SL., Paredes, SH., Yourstone, S., Gehring, J., Malfatti, S., et al. (2012). Defining the core Arabidopsis thaliana root microbiome. *Nature* 488, 86-90.

McMurdie, PJ., Holmes, S. (2013). phyloseq: an R package for reproducible interactive analysis and graphics of microbiome census data. *PLoS One* 8, e61217.

Newman, ME. Modularity and community structure in networks. (2006). *Proc. Natl. Acad. Sci. U.S.A* 103, 8577-8582.

Peiffer, JA., Spor, A., Koren, O., Jin, Z., Tringe, SG., Dangl, JL., *et al*. (2013). Diversity and heritability of the maize rhizosphere microbiome under field conditions. *Proc. Natl. Acad. Sci. U.S.A.* 110, 6548-6553.

Rognes, T., Flouri, T., Nichols, B., Quince, C., Mahé, F. (2016). VSEARCH: a versatile open source tool for metagenomics. *PeerJ.* 4, e2584.

Shannon, P., Markiel, A., Ozier, O., Baliga, N.S., Wang, J.T., Ramage, D., et al. (2003). Cytoscape: a software environment for integrated models of biomolecular interaction networks. *Genome Res*. 13, 2498-2504.

Wagner, MR., Lundberg, DS., Tijana, G., Tringe, SG., Dangl, JL., Mitchell-Olds, T. (2016). Host genotype and age shape the leaf and root microbiomes of a wild perennial plant. *Nat. Commun.* 7, 12151.

Zhou, J., Deng, Y., Luo, F., He, Z., Tu, Q., Zhi, X. (2010). Functional molecular ecological networks. *MBio* 1, e00169-00110.


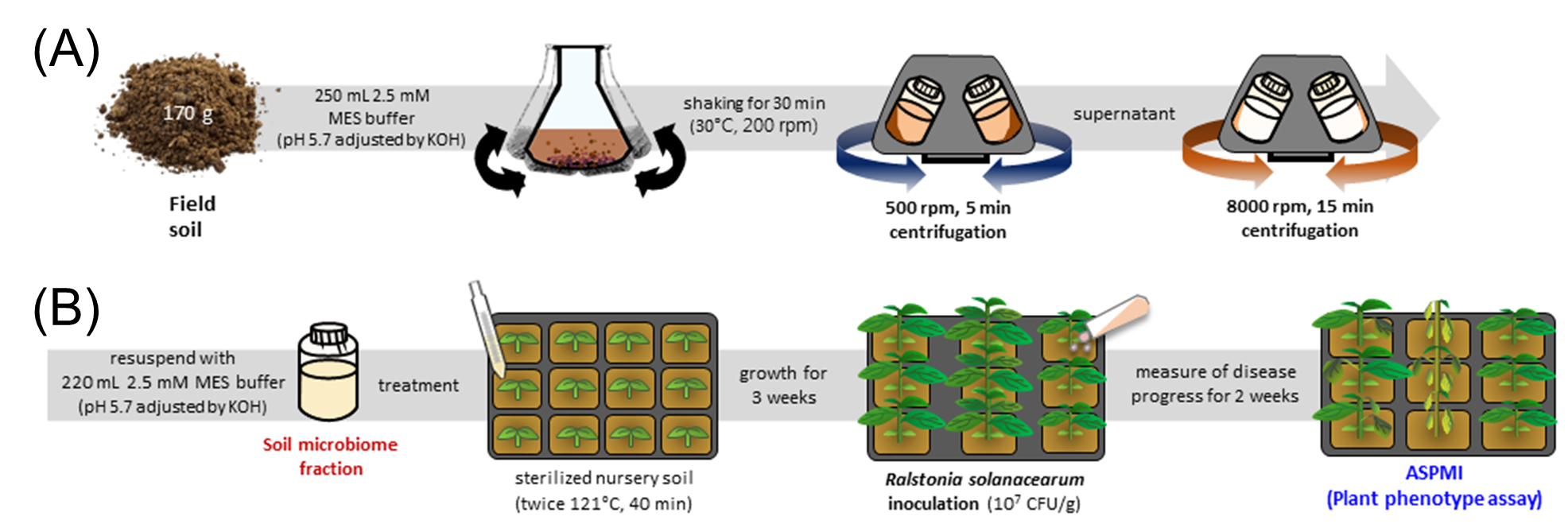


Supplementary Figure S1. Fractionation of microbiotas from natural bulk soils and development of an analysis system for plant-microbiome interaction (ASPMI). The soil microbial fraction (MF) was isolated from the field soils (A). The planted tomato seedlings were treated with soil MF and were grown for 3 weeks before *Ralstonia solanacearum* inoculation (B). This specific example represents the assay to investigate disease progress of bacterial wilt by *R. solanacearum*.


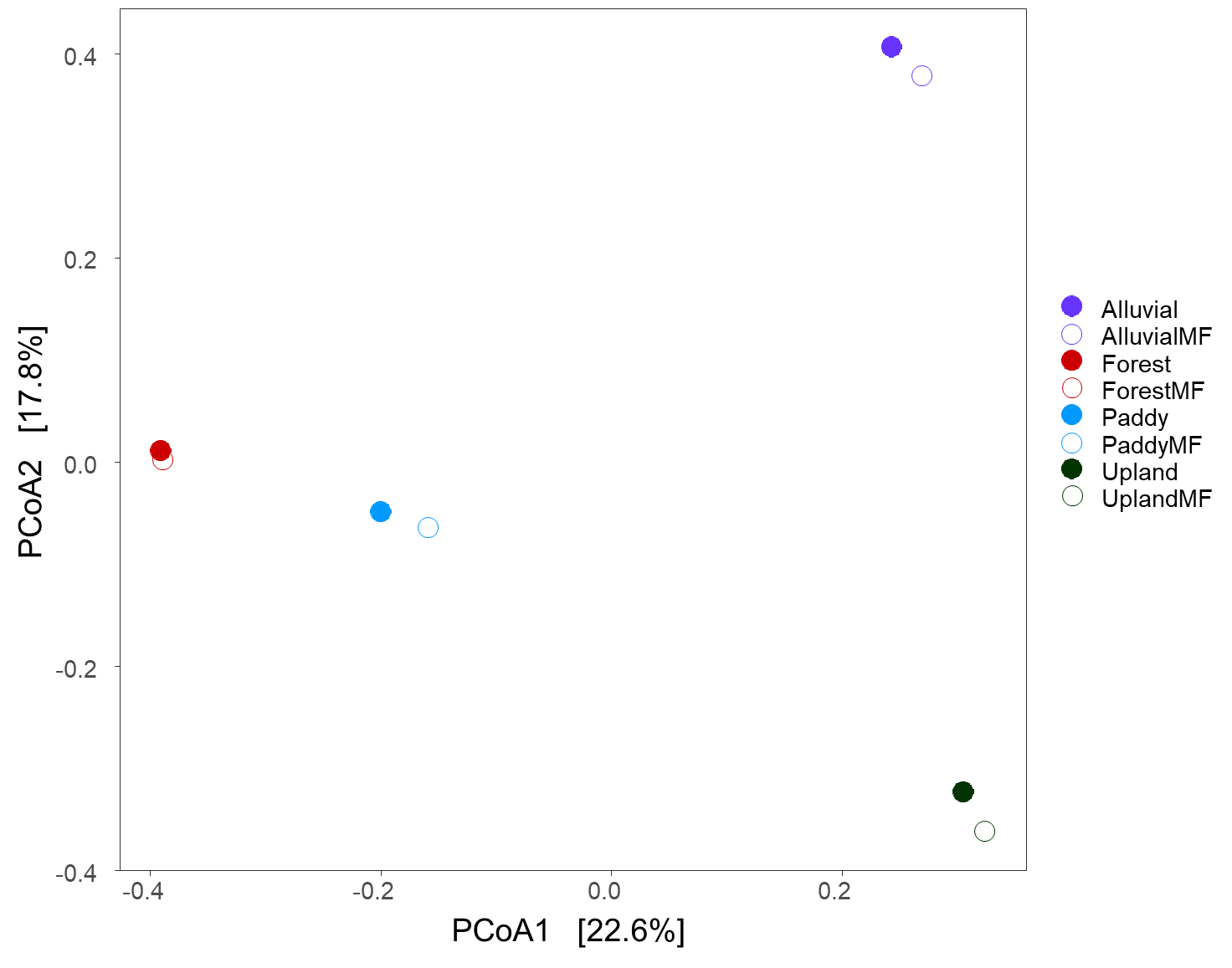


Supplementary Figure S2. Unconstrained principal coordinate analysis with the pairwise community comparison by Bray-Curtis dissimilarity measure of the microbial communities of 4 different natural soils and their corresponding microbial fraction (MF).


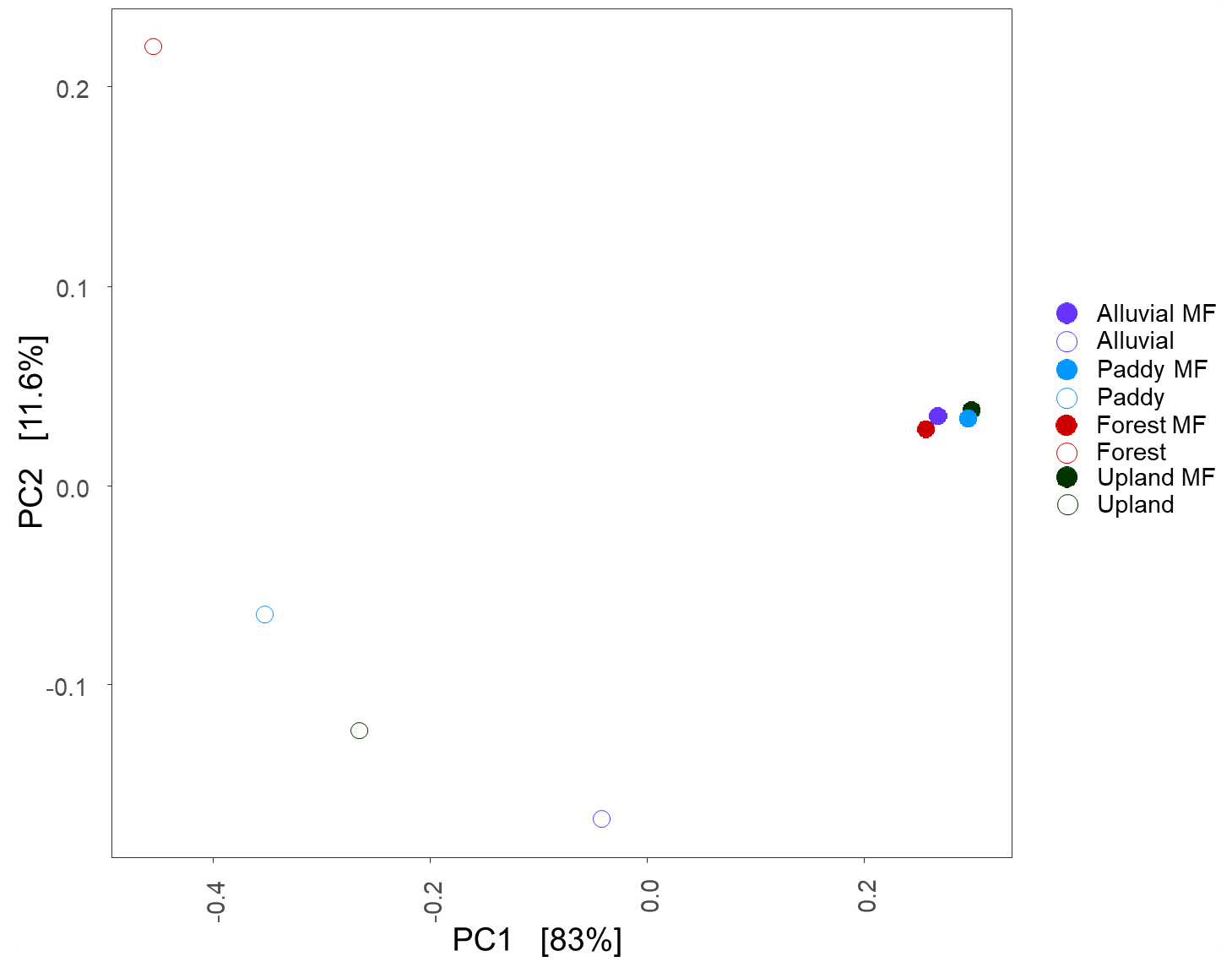


Supplementary Figure S3. Principle component analysis of correlation using 13 physical and chemical properties of the eight soils.
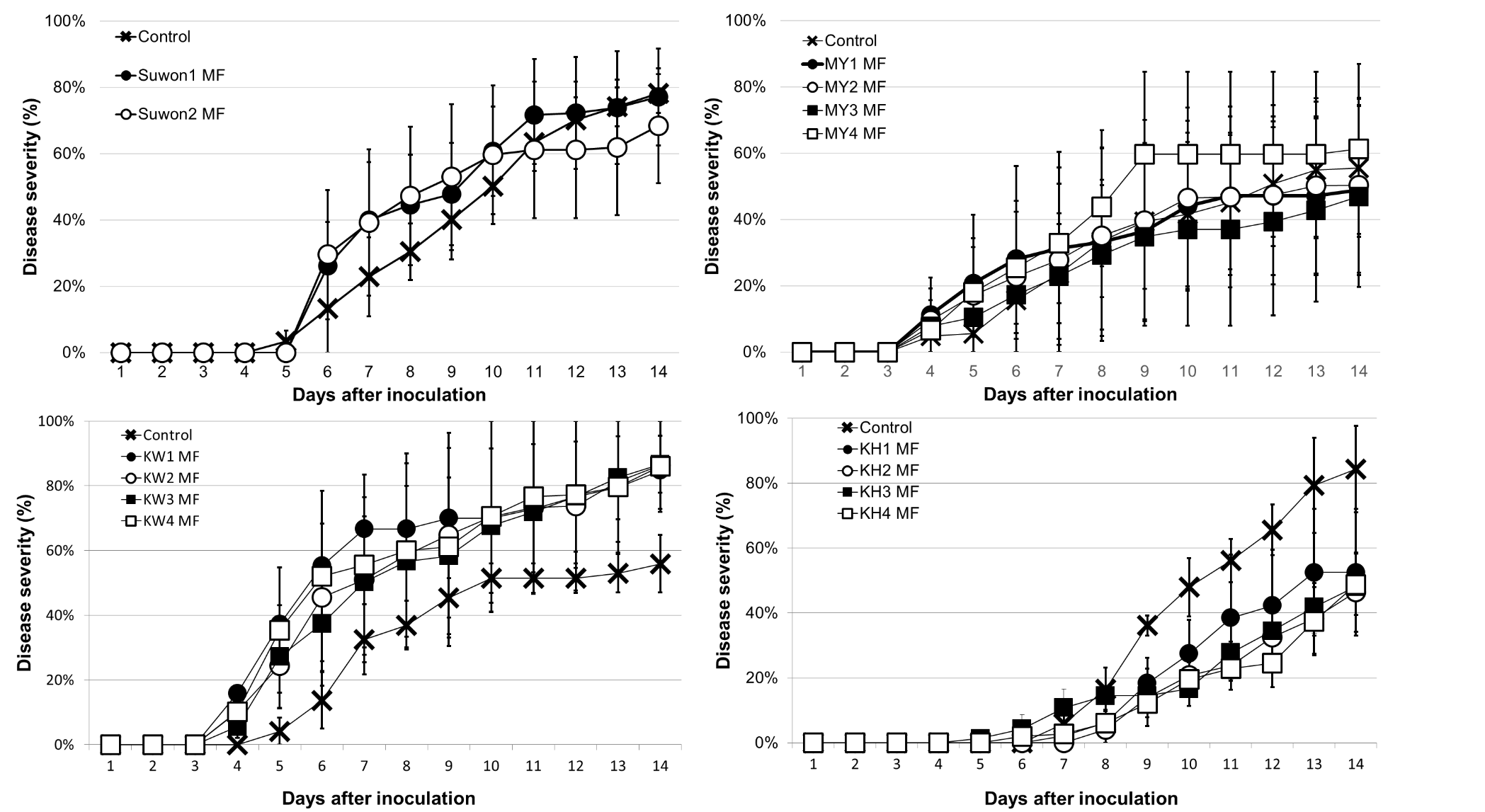


Supplementary Figure S4. Bacterial wilt (BW) disease progression in tomato cultivars treated with soil microbial fractions (MFs) evaluated by ASPMI method. BW disease progression in the 14 different soil MFs-treated the resistant cultivar, Hawaii 7996 inoculated with *R. solanacearum* SL341 strain.


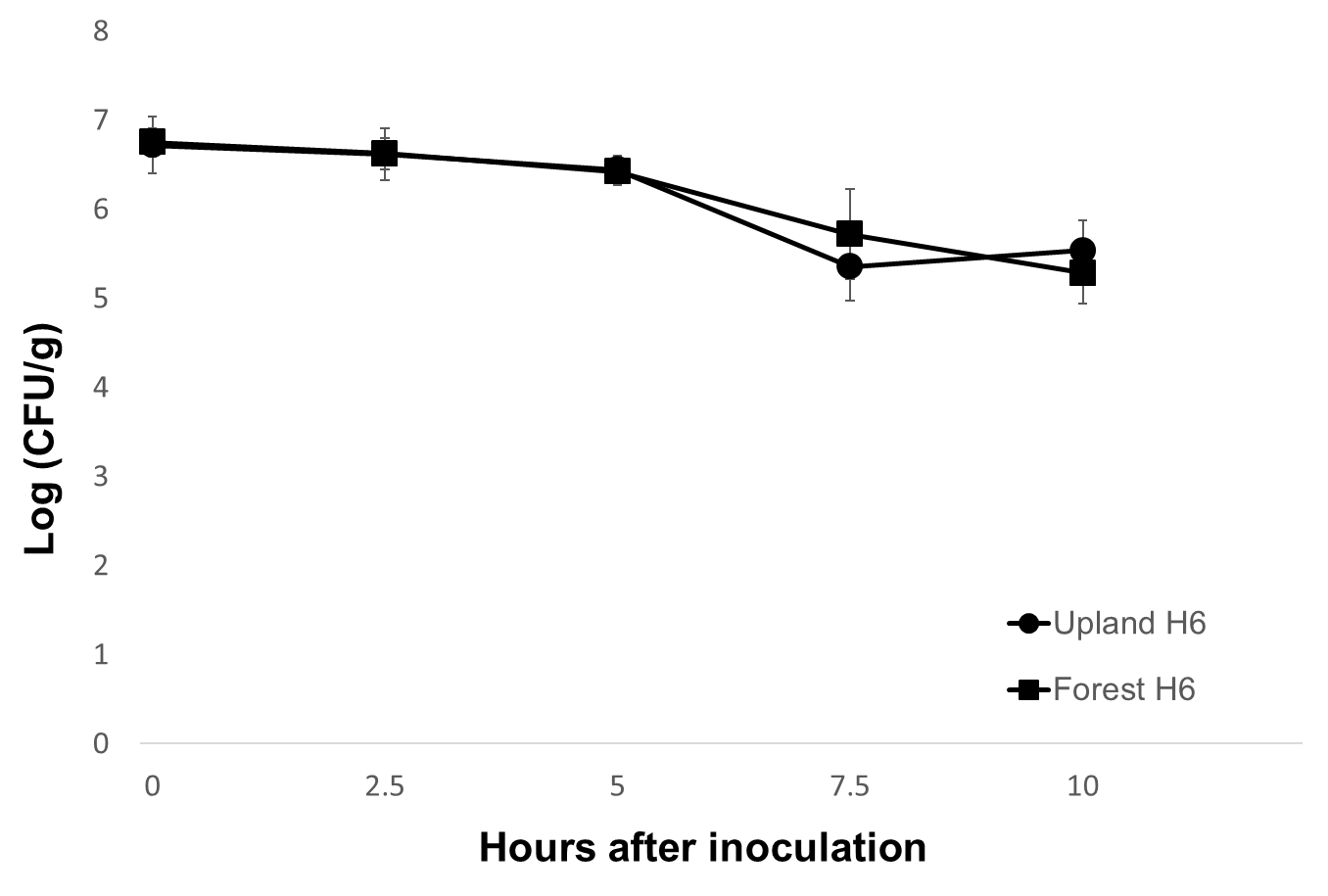


Supplementary Figure S5. Effect of microbial fraction (MF) isolated from tomato cultivar Hawaii 7996 rhizosphere on the bacterial growth of *R. solanacearum* SL341. Upland H6 and Forest H6 represent each MF from Hawaii 7996 rhizosphere previously treated with upland MF and forest MF grown for 4 weeks, respectively. Vertical bars indicate the standard deviations from replicates.


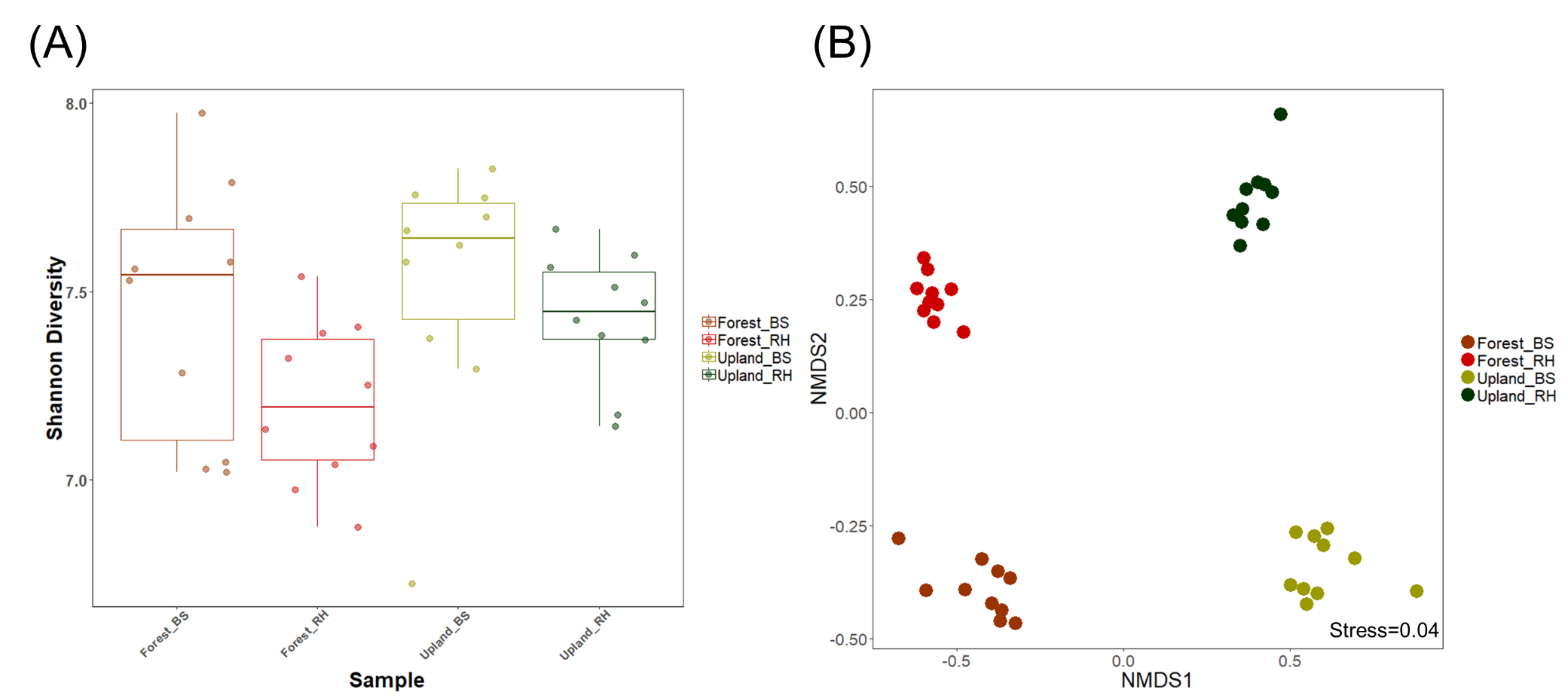


Supplementary Figure S6. Microbial community comparison in tomato rhizosphere and bulk soil. (A) Alpha-diversity analysis of bulk soil and rhizosphere bacterial community of resistant cultivar between forest and upland MF under ASPMI. Shannon diversity index (*H’*) was calculated to estimate taxonomic parameter. (B) Bacterial community comparison of resistant cultivar treated with forest and upland MF under ASPMI. Non-metric multi-dimensional scaling based on the Bray-Curtis dissimilarity measures visualized patterns of distribution of the two rhizosphere microbiotas in the resistant cultivar. Forest_BS, bulk soil treated with forest MF; Forest_RH, rhizosphere treated with forest MF; Upland_BS, bulk soil treated with upland MF; Upland_RH, rhizosphere treated with upland MF;


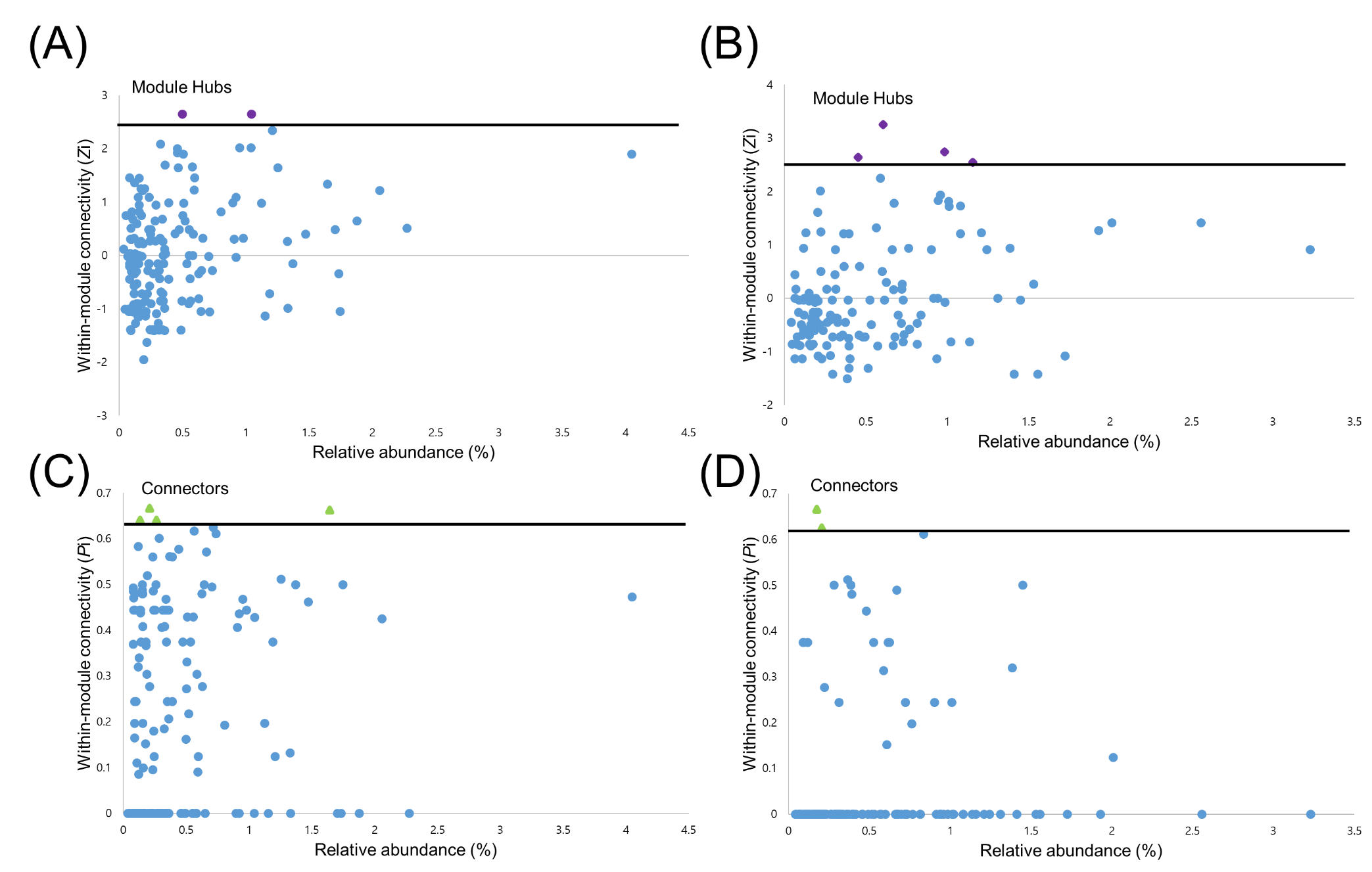


Supplementary Figure S7. The relative abundance of module hubs (purple) in the Hawaii 7996 rhizosphere treated with upland MF (A) and forest MF (B) and module connectors (green) in the rhizosphere treated with upland MF (C) and forest MF (D). Black line indicate the threshold above which nodes are classified module hubs (A and B) based on within-module connectivity (*Zi* > 2.5), or as module connectors (C and D) based on among-module connectivity (*Pi* > 0.62)

Supplementary Table S1. List of the 18 soils

| Sample | Type of soil | GPS |
| --- | --- | --- |
| Suwon 1 | Paddy soil under rice (Hwaseonchal) cultivation | 37°16’25’’N 126°59’35’’E |
| Suwon 2 | Paddy soil under rice (Seolremi) cultivation | 37°16’26’’N 126°59’35’’E |
| MY1 | Paddy soil under rice (Jinsumi) cultivation | 35°29’35.858”N 128°44’32.864”E |
| MY2 | Upland soil under red bean cultivation | 35°29’29.705”N 128°44’20.889”E |
| MY3 | Upland soil under soybean cultivation | 35°29’32.575”N 128°44’21.053”E |
| MY4 | Upland soil under sesame cultivation | 35°29’37.478”N 128°44’19.406”E |
| KW1 | Alpine crop field (potato) | 35°40’41.437”N 128°45’00.000”E |
| KW2 | Alpine crop field (cabbage) | 35°40’41.080”N 128°44’59.176”E |
| KW3 | Alpine crop field (corn) | 35°40’41.568”N 128°44’58.792”E |
| KW4 | Alpine crop field (pasture) | 37°41’03.066”N 128°44’04.574”E |
| KH1 | Clay soil more than 10 years under tomato cultivation | 35°12’28.677”N 128°50’15.857”E |
| KH2 | Sandy soil under tomato (Jjapjjal) cultivation | 35°10’07.558”N 128°55’14.905”E |
| KH3 | Loess soil | 35°17’07.798”N 128°52’36.317”E |
| KH4 | Terra alba soil | 35°16’57.705”N 128°52’40.382”E |
| Upland | Upland soil under tomato cultivation | 35.239°N 128.978°E |
| Paddy | Paddy soil under rice | 35°14’22”N 128°58’43”E |
| Forest | Forest soil under oak tree | 35°6’51”N 128°58’4”E |
| Alluvial | Alluvial soil | 35°5.28”N 128°56.479”E |

Supplementary Table S2. Physicochemical properties of original field soils and sterilized commercial nursery soil inoculated by soil microbiome fractions (MFs) from 4 different soils.

|  | pH | Organic matter (**%)** | N (**mg/kg)** | Ca (**mg/kg)** | Mg (**mg/kg)** | K (**mg/kg)** | Na (**mg/kg)** | P_2_O_5_ (**mg/kg)** | Fe (**mg/kg)** | Mn (**mg/kg)** | S (**mg/kg)** | Cu (**mg/kg)** | Zn (**mg/kg)** |
| --- | --- | --- | --- | --- | --- | --- | --- | --- | --- | --- | --- | --- | --- |
| **Upland** | 6.9 | 1.8 | 191.627 | 2597.37 | 346.9 | 61.66 | 413.06 | 396.74 | 25356.23 | 435.34 | 426.92 | 20 | 157.2 |
| **Paddy** | 5.6 | 2.52 | 4.53 | 1377.55 | 225.69 | 109.71 | 128.55 | 72.74 | 30331.9 | 385.01 | 377.73 | 17.4 | 77.9 |
| **Forest** | 4.7 | 7.62 | 10.981 | 709.81 | 196.83 | 109.82 | 26.12 | 19.6 | 61507.56 | 629.63 | 367.74 | 24.2 | 126 |
| **Alluvial** | 8.4 | 1.58 | 31 | 20332.14 | 3592.74 | 1841.31 | 361.16 | 832.09 | 22269.31 | 883.53 | 866.6 | 5.4 | 46.2 |
| **Upland MF** | 5.7 | 44.47 | 1233.4 | 8653.11 | 7142.27 | 10782.71 | 1433.72 | 1117.25 | 9997.15 | 198.17 | 690.13 | 10.1 | 32.8 |
| **Paddy MF** | 5.9 | 39.86 | 975.4 | 8636.51 | 6912.34 | 10732.25 | 1578.32 | 1133.74 | 10106.21 | 197.38 | 765.3 | 9.4 | 32.6 |
| **Forest MF** | 5.9 | 41.21 | 1067.4 | 8696.78 | 7685.04 | 11430.65 | 1453.59 | 1234 | 12341.01 | 231.56 | 709.37 | 11.5 | 39.7 |
| **Alluvial MF** | 5.8 | 37.98 | 1126.7 | 8702.11 | 7551.51 | 12067.21 | 1439.78 | 1202.16 | 11734.05 | 352.54 | 701.72 | 9.2 | 35.8 |

Supplementary Table S3. List of *R. solanacearum* strains

| Bacterial strains^a^ | Other collection number^b^ | Host (Geographical origin) | Race | Biovar | Phylotype | Sequevar |
| --- | --- | --- | --- | --- | --- | --- |
| *R. solanacearum* GMI1000 | N/A | Tomato (Guyana) | 1 | 3 | I | 18 |
| *R. solanacearum* SL341 | KACC10709 | Tomato (South Korea) | 1 | 3 | I | N/A |
| *R. solanacearum* LMG2305 | NCPPB 909 | Potato (Egypt) | 1 | 3 | III | N/A |
| *R. solanacearum* LMG5839 | NCPPB 1579 | Ginger (USA) | 1 | 4 | I | N/A |
| *R. solanacearum* LMG17138 | NCPPB 3866 | Potato (Brazil) | 1 | 1 | II | N/A |
| *R. solanacearum* LMG17139 | NCPPB 1584 | Potato (Cyprus) | 3 | 2 | II | 1 |

^a^R, Ralstonia, LMG, Belgian Co-ordinated Collections of Micro-organisms;

^b^ N/A, Not applicable; KACC, Korean Agricultural Culture Collection; NCPPB, The National Collection of Plant Pathogenic Bacteria

Supplementary Table S4. Differential relative abundance of microbial OTUs between the Hawaii 7996 rhizosphere microbiota under the ASPMI treated with 2 different soil MFs (upland and forest). Features were considered significant if their false discovery rate-corrected *p*-value was less than or equal to 0.0001, and the absolute value of the log2 fold change (groups compared: rhizosphere microbiota treated by upland MF over rhizosphere microbiota treated by forest MF) was greater than or equal to 1.

| Phylum | Class | Order | Family | Genus | Species | baseMean | log2FoldChange | padj |
| --- | --- | --- | --- | --- | --- | --- | --- | --- |
| Verrucomicrobia | [Pedosphaerae] | [Pedosphaerales] | auto67_4W |  |  | 283.1212 | 11.6218 | 9.54E-27 |
| Verrucomicrobia | Opitutae | Opitutales | Opitutaceae | Opitutus |  | 261.6512 | 11.50797 | 5.22E-24 |
| Proteobacteria | Betaproteobacteria | Burkholderiales | Burkholderiaceae | Salinispora |  | 162.5704 | 10.82137 | 3.56E-23 |
| Proteobacteria | Betaproteobacteria | Burkholderiales | Oxalobacteraceae | Cupriavidus |  | 125.5286 | 10.44835 | 1.89E-20 |
| Proteobacteria | Gammaproteobacteria | Pseudomonadales | Pseudomonadaceae | Pseudomonas | Unclassified | 121.9458 | 10.40657 | 8.90E-22 |
| Proteobacteria | Betaproteobacteria | Burkholderiales |  |  |  | 100.1652 | 10.12283 | 1.30E-20 |
| Proteobacteria | Gammaproteobacteria | Xanthomonadales | Xanthomonadaceae | Luteimonas |  | 94.74499 | 10.04273 | 3.87E-18 |
| Proteobacteria | Betaproteobacteria | Burkholderiales | Oxalobacteraceae | Ralstonia |  | 89.39536 | 9.958668 | 7.51E-19 |
| Bacteroidetes | [Saprospirae] | [Saprospirales] | Chitinophagaceae |  |  | 85.6559 | 9.896969 | 9.13E-19 |
| Planctomycetes | Planctomycetia | Gemmatales | Gemmataceae | Gemmata |  | 81.90778 | 9.832314 | 1.43E-19 |
| Planctomycetes | Planctomycetia | Planctomycetales | Planctomycetaceae | Planctomyces |  | 79.22051 | 9.784299 | 6.56E-19 |
| Verrucomicrobia | Opitutae | Opitutales | Opitutaceae |  |  | 79.17755 | 9.783669 | 9.97E-20 |
| Bacteroidetes | [Saprospirae] | [Saprospirales] | Chitinophagaceae | Sediminibacterium |  | 71.79644 | 9.642032 | 3.06E-18 |
| Bacteroidetes | [Saprospirae] | [Saprospirales] | Chitinophagaceae |  |  | 68.34206 | 9.571278 | 4.08E-18 |
| Bacteroidetes | Sphingobacteriia | Sphingobacteriales | |  |  | 67.83123 | 9.560368 | 3.22E-18 |
| Bacteroidetes | Cytophagia | Cytophagales | Cytophagaceae |  |  | 66.53152 | 9.532232 | 4.76E-18 |
| Proteobacteria | Betaproteobacteria | Methylophilales | Methylophilaceae | Methylobacillus |  | 64.53912 | 9.488545 | 5.19E-16 |
| Planctomycetes | Planctomycetia | Pirellulales | Pirellulaceae |  |  | 59.87344 | 9.380376 | 4.08E-18 |
| Proteobacteria | Gammaproteobacteria | Xanthomonadales | Sinobacteraceae |  |  | 54.02084 | 9.231909 | 4.10E-11 |
| Proteobacteria | Alphaproteobacteria | Rhizobiales | Bradyrhizobiaceae | Bosea | genosp. | 53.30283 | 9.212622 | 4.50E-17 |
| TM6 | SJA-4 |  |  |  |  | 48.80447 | 9.085777 | 4.41E-15 |
| Planctomycetes | Planctomycetia | Gemmatales | Isosphaeraceae | Singulisphaera |  | 48.6757 | 9.081663 | 4.96E-08 |
| FBP |  |  |  |  |  | 48.66418 | 9.081466 | 9.69E-17 |
| Planctomycetes | Planctomycetia | Pirellulales | Pirellulaceae |  |  | 46.52766 | 9.016468 | 1.01E-16 |
| TM7 | TM7-1 |  |  |  |  | 45.62817 | 8.98868 | 1.85E-15 |
| Proteobacteria | Alphaproteobacteria | Sphingomonadales | Sphingomonadaceae | Sphingobium |  | 45.18417 | 8.97479 | 2.22E-15 |
| Verrucomicrobia | Opitutae | Opitutales | Opitutaceae |  |  | 44.65004 | 8.957301 | 2.94E-15 |
| Proteobacteria | Alphaproteobacteria | Ellin329 |  |  |  | 43.65533 | 8.924562 | 7.04E-16 |
| Verrucomicrobia | [Pedosphaerae] | [Pedosphaerales] |  |  |  | 43.62724 | 8.923193 | 5.74E-15 |
| Proteobacteria | Gammaproteobacteria | Xanthomonadales | Xanthomonadaceae | Rhodanobacter |  | 42.7402 | 8.894277 | 8.10E-11 |
| Planctomycetes | Planctomycetia | Gemmatales | Gemmataceae | Gemmata |  | 42.50011 | 8.885814 | 2.50E-16 |
| Proteobacteria | Gammaproteobacteria | Legionellales | Coxiellaceae | Aquicella |  | 41.17339 | 8.840189 | 1.24E-09 |
| Proteobacteria | Alphaproteobacteria | Ellin329 |  |  |  | 38.81729 | 8.755199 | 1.63E-15 |
| Proteobacteria | Betaproteobacteria | Neisseriales | Neisseriaceae | Chromobacterium |  | 38.76292 | 8.753068 | 9.17E-05 |
| Planctomycetes | Planctomycetia | Gemmatales | Isosphaeraceae |  |  | 38.47003 | 8.742351 | 5.97E-06 |
| Chlamydiae | Chlamydiia | Chlamydiales |  |  |  | 37.8115 | 8.716424 | 1.93E-13 |
| Proteobacteria | Gammaproteobacteria | Legionellales | Coxiellaceae | Aquicella |  | 37.66806 | 8.712026 | 5.58E-06 |
| Proteobacteria | Gammaproteobacteria | Xanthomonadales | Sinobacteraceae | Nevskia |  | 36.21999 | 8.655014 | 5.55E-10 |
| Bacteroidetes | Sphingobacteriia | Sphingobacteriales | |  |  | 36.19464 | 8.654508 | 2.44E-14 |
| Proteobacteria | Gammaproteobacteria | Legionellales | Legionellaceae | Legionella |  | 34.32529 | 8.577925 | 2.04E-07 |
| Actinobacteria | Actinobacteria | Actinomycetales | Nocardioidaceae | Pimelobacter |  | 33.5495 | 8.544622 | 1.14E-09 |
| Acidobacteria | Solibacteres | Solibacterales |  |  |  | 33.03919 | 8.522088 | 5.74E-15 |
| Bacteroidetes | [Saprospirae] | [Saprospirales] | Chitinophagaceae |  |  | 31.96825 | 8.475328 | 5.54E-10 |
| Proteobacteria | Alphaproteobacteria | Rhizobiales |  |  |  | 31.80982 | 8.468382 | 5.55E-10 |
| OD1 | ZB2 |  |  |  |  | 31.16222 | 8.438121 | 5.16E-07 |
| Proteobacteria | Gammaproteobacteria | Xanthomonadales | Xanthomonadaceae |  |  | 31.06961 | 8.434587 | 1.85E-07 |
| Planctomycetes | Planctomycetia | Pirellulales | Pirellulaceae | A17 |  | 31.03571 | 8.432223 | 3.05E-07 |
| Proteobacteria | Betaproteobacteria | Rhodocyclales | Rhodocyclaceae | Uliginosibacterium |  | 30.59728 | 8.411883 | 9.38E-14 |
| Proteobacteria | Alphaproteobacteria | Rhodobacterales | Hyphomonadaceae |  |  | 29.53542 | 8.360372 | 1.72E-13 |
| Proteobacteria | Gammaproteobacteria | Unclassified | Unclassified | Unclassified | Unclassified | 28.50563 | 8.310009 | 4.24E-13 |
| Proteobacteria | Gammaproteobacteria | Xanthomonadales | Xanthomonadaceae |  |  | 28.26426 | 8.297684 | 1.33E-13 |
| Bacteroidetes | [Saprospirae] | [Saprospirales] | Chitinophagaceae | Niabella |  | 25.79875 | 8.165817 | 1.79E-05 |
| Verrucomicrobia | [Pedosphaerae] | [Pedosphaerales] | Ellin517 |  |  | 25.53179 | 8.151388 | 1.80E-09 |
| Bacteroidetes | Cytophagia | Cytophagales | Cytophagaceae | Sporocytophaga |  | 25.26131 | 8.13547 | 2.03E-13 |
| OD1 | SM2F11 |  |  |  |  | 24.80921 | 8.108909 | 6.96E-12 |
| Bacteroidetes | [Saprospirae] | [Saprospirales] | Chitinophagaceae |  |  | 24.12978 | 8.0685 | 1.48E-08 |
| Proteobacteria | Alphaproteobacteria | Rhizobiales | Hyphomicrobiaceae | Rhodoplanes |  | 24.07769 | 8.066441 | 5.71E-07 |
| Proteobacteria | Alphaproteobacteria | Rhizobiales | Beijerinckiaceae | Beijerinckia |  | 23.97324 | 8.060117 | 1.09E-13 |
| Proteobacteria | Gammaproteobacteria | Xanthomonadales | Sinobacteraceae |  |  | 23.39394 | 8.025116 | 6.52E-07 |
| Bacteroidetes | [Saprospirae] | [Saprospirales] | Chitinophagaceae |  |  | 22.79701 | 7.988032 | 8.76E-07 |
| Proteobacteria | Gammaproteobacteria | Xanthomonadales | Xanthomonadaceae | Thermomonas |  | 22.7176 | 7.98106 | 2.23E-08 |
| Proteobacteria | Gammaproteobacteria | Xanthomonadales | Xanthomonadaceae | Dokdonella |  | 22.68505 | 7.98034 | 2.12E-05 |
| Planctomycetes | Planctomycetia | Planctomycetales | Planctomycetaceae | Planctomyces |  | 22.11665 | 7.942932 | 1.48E-08 |
| Verrucomicrobia | Verrucomicrobiae | Verrucomicrobiales | Verrucomicrobiaceae |  |  | 20.95885 | 7.865335 | 1.18E-11 |
| Bacteroidetes | [Saprospirae] | [Saprospirales] | Chitinophagaceae | Chitinophaga |  | 20.59243 | 7.841414 | 1.97E-06 |
| Chlamydiae | Chlamydiia | Chlamydiales | Rhabdochlamydiaceae | Candidatus Rhabdochlamydia | | 20.50109 | 7.834526 | 1.09E-10 |
| Chlorobi |  |  |  |  |  | 20.32696 | 7.821957 | 1.58E-06 |
| Proteobacteria | Alphaproteobacteria | Rhizobiales | Bradyrhizobiaceae |  |  | 20.30251 | 7.820922 | 7.09E-09 |
| Verrucomicrobia | [Pedosphaerae] | [Pedosphaerales] | Ellin517 |  |  | 20.10493 | 7.806429 | 1.70E-08 |
| Acidobacteria | Acidobacteriia | Acidobacteriales | Acidobacteriaceae |  |  | 19.83714 | 7.786177 | 2.11E-06 |
| Proteobacteria | Gammaproteobacteria | Xanthomonadales | Sinobacteraceae | Unclassified | Unclassified | 19.22326 | 7.741819 | 1.78E-08 |
| TM6 | SJA-4 |  |  |  |  | 18.99028 | 7.723932 | 2.07E-06 |
| Verrucomicrobia | [Spartobacteria] | [Chthoniobacterales] | [Chthoniobacteraceae] | Ellin506 |  | 18.45568 | 7.682477 | 2.52E-06 |
| Armatimonadetes | [Fimbriimonadia] | [Fimbriimonadales] | [Fimbriimonadaceae] | Fimbriimonas |  | 18.38102 | 7.676949 | 1.98E-06 |
| Verrucomicrobia | [Pedosphaerae] | [Pedosphaerales] |  |  |  | 18.32147 | 7.672662 | 2.16E-08 |
| OD1 | ZB2 |  |  |  |  | 17.63549 | 7.616453 | 9.98E-05 |
| Proteobacteria | Alphaproteobacteria | Rhodospirillales | Rhodospirillaceae |  |  | 17.52808 | 7.60899 | 3.69E-05 |
| Proteobacteria | Deltaproteobacteria | Myxococcales | 0319-6G20 |  |  | 17.07393 | 7.570915 | 1.81E-08 |
| Proteobacteria | Deltaproteobacteria |  |  |  |  | 16.94528 | 7.559728 | 4.30E-06 |
| Acidobacteria | Solibacteres | Solibacterales | Solibacteraceae |  |  | 16.4059 | 7.512615 | 2.99E-06 |
| Verrucomicrobia | Opitutae | Opitutales | Opitutaceae | Opitutus |  | 16.13022 | 7.48733 | 5.58E-06 |
| Verrucomicrobia | [Pedosphaerae] | [Pedosphaerales] |  |  |  | 15.9546 | 7.473714 | 2.62E-06 |
| Bacteroidetes | [Saprospirae] | [Saprospirales] | Chitinophagaceae |  |  | 15.76465 | 7.455554 | 4.72E-06 |
| Bacteroidetes | Cytophagia | Cytophagales | Cytophagaceae | Dyadobacter |  | 15.44268 | 7.426386 | 5.36E-08 |
| Chlamydiae | Chlamydiia | Chlamydiales | Unclassified | Unclassified | Unclassified | 15.13572 | 7.397129 | 6.84E-05 |
| Planctomycetes | Planctomycetia | Planctomycetales | Planctomycetaceae | Planctomyces |  | 14.57334 | 7.341794 | 8.03E-05 |
| Proteobacteria | Alphaproteobacteria | Rhodospirillales | Rhodospirillaceae | Phaeospirillum | fulvum | 14.55348 | 7.339126 | 9.56E-06 |
| Proteobacteria | Deltaproteobacteria | Bdellovibrionales | Bdellovibrionaceae | Bdellovibrio |  | 14.48693 | 7.333461 | 0.0007 |
| Planctomycetes | Planctomycetia | Pirellulales | Pirellulaceae |  |  | 14.08659 | 7.294096 | 6.83E-06 |
| Proteobacteria | Deltaproteobacteria | Myxococcales | Myxococcaceae |  |  | 13.48088 | 7.229761 | 7.07E-06 |
| Chlamydiae | Chlamydiia | Chlamydiales | Parachlamydiaceae | Unclassified | Unclassified | 13.44781 | 7.226633 | 0.000665 |
| Proteobacteria | Betaproteobacteria | Burkholderiales | Burkholderiaceae | Burkholderia | tuberum | 13.42463 | 7.223594 | 0.000716 |
| Proteobacteria | Betaproteobacteria | Burkholderiales | Alcaligenaceae |  |  | 13.12294 | 7.190419 | 0.00099 |
| Gemmatimonadetes | Gemmatimonadetes | Ellin5290 |  |  |  | 12.70399 | 7.145192 | 5.90E-06 |
| OD1 | ZB2 |  |  |  |  | 12.27082 | 7.093967 | 1.90E-05 |
| Proteobacteria | Deltaproteobacteria | Myxococcales | Haliangiaceae |  |  | 12.26219 | 7.093484 | 8.11E-06 |
| Proteobacteria | Deltaproteobacteria | Bdellovibrionales | Bdellovibrionaceae | Bdellovibrio |  | 12.20638 | 7.087455 | 1.20E-05 |
| Proteobacteria | Gammaproteobacteria | Legionellales | Legionellaceae | Tatlockia |  | 12.03362 | 7.065504 | 1.22E-05 |
| Proteobacteria | Gammaproteobacteria | Legionellales | Unclassified | Unclassified | Unclassified | 11.95004 | 7.056576 | 2.06E-05 |
| Unclassified | Unclassified | Unclassified | Unclassified | Unclassified | Unclassified | 11.87017 | 7.046548 | 0.000167 |
| TM6 | SJA-4 |  |  |  |  | 11.0153 | 6.939192 | 1.63E-06 |
| Gemmatimonadetes | Gemmatimonadetes | Ellin5290 |  |  |  | 10.871 | 6.920042 | 2.52E-07 |
| Planctomycetes | Planctomycetia | Gemmatales | Gemmataceae | Gemmata |  | 10.81296 | 6.912017 | 0.000177 |
| Planctomycetes | Planctomycetia | Planctomycetales | Planctomycetaceae | Planctomyces |  | 10.70057 | 6.896792 | 1.41E-05 |
| Proteobacteria | Alphaproteobacteria | Unclassified | Unclassified | Unclassified | Unclassified | 10.36024 | 6.84882 | 1.61E-09 |
| Proteobacteria | Alphaproteobacteria | Unclassified | Unclassified | Unclassified | Unclassified | 10.31224 | 6.843426 | 0.000223 |
| Verrucomicrobia | Verrucomicrobiae | Verrucomicrobiales | Verrucomicrobiaceae |  |  | 9.825514 | 6.772244 | 1.72E-06 |
| Proteobacteria | Gammaproteobacteria | Legionellales | Unclassified | Unclassified | Unclassified | 9.516718 | 6.726987 | 0.000361 |
| TM6 | SJA-4 |  |  |  |  | 41.4492 | 6.625777 | 1.60E-07 |
| Planctomycetes | Planctomycetia | Pirellulales | Pirellulaceae | Pirellula |  | 8.737086 | 6.605341 | 0.000305 |
| Proteobacteria | Gammaproteobacteria | Pseudomonadales | Moraxellaceae | Perlucidibaca |  | 69.87615 | 6.533092 | 1.59E-06 |
| Bacteroidetes | [Saprospirae] | [Saprospirales] | Chitinophagaceae | Flavisolibacter |  | 8.050942 | 6.486461 | 0.000383 |
| Proteobacteria | Gammaproteobacteria | Legionellales |  |  |  | 6.753599 | 6.233457 | 8.78E-05 |
| Proteobacteria | Betaproteobacteria | Burkholderiales | Oxalobacteraceae | Cupriavidus |  | 69.45922 | 6.144449 | 2.34E-05 |
| Planctomycetes | Phycisphaerae | WD2101 |  |  |  | 6.279758 | 6.124373 | 1.14E-05 |
| Proteobacteria | Betaproteobacteria | Burkholderiales |  |  |  | 72.28189 | 5.601179 | 0.000304 |
| Proteobacteria | Alphaproteobacteria | Caulobacterales | Caulobacteraceae | Asticcacaulis | biprosthecium | 183.0872 | 2.54757 | 0.000435 |
| Firmicutes | Bacilli | Bacillales | Alicyclobacillaceae | Alicyclobacillus |  | 729.1735 | 1.652287 | 0.000129 |
| Bacteroidetes | Sphingobacteriia | Sphingobacteriales | Sphingobacteriaceae |  |  | 149.6062 | -1.65669 | 0.000271 |
| Planctomycetes | Phycisphaerae | WD2101 |  |  |  | 43.11172 | -4.4156 | 0.000497 |
| Chlamydiae | Chlamydiia | Chlamydiales | Parachlamydiaceae |  |  | 42.97925 | -5.42673 | 1.72E-05 |
| TM7 | TM7-3 | EW055 |  |  |  | 124.1241 | -5.50881 | 1.21E-05 |
| Planctomycetes | Planctomycetia | Pirellulales | Pirellulaceae |  |  | 5.61398 | -6.03337 | 0.000158 |
| Bacteroidetes | [Saprospirae] | [Saprospirales] | Chitinophagaceae | Flavisolibacter |  | 6.496134 | -6.24344 | 0.000681 |
| Acidobacteria | Acidobacteriia | Acidobacteriales | Acidobacteriaceae |  |  | 164.0554 | -6.2852 | 1.88E-07 |
| WPS-2 |  |  |  |  |  | 7.18681 | -6.38837 | 0.000548 |
| Planctomycetes | Planctomycetia | Gemmatales | Gemmataceae |  |  | 7.518396 | -6.4548 | 0.000435 |
| Gemmatimonadetes | Gemmatimonadetes | Ellin5290 |  |  |  | 8.131392 | -6.56851 | 0.000332 |
| Verrucomicrobia | [Spartobacteria] | [Chthoniobacterales] | [Chthoniobacteraceae] | Candidatus Xiphinematobacter | | 8.688316 | -6.66335 | 0.000284 |
| Planctomycetes | Phycisphaerae | WD2101 |  |  |  | 9.674114 | -6.81834 | 0.000209 |
| Chloroflexi | Ktedonobacteria | Thermogemmatisporales | Thermogemmatisporaceae |  |  | 10.39458 | -6.9215 | 0.000175 |
| Acidobacteria | Acidobacteriia | Acidobacteriales | Acidobacteriaceae | Edaphobacter | Unclassified | 10.47311 | -6.93281 | 1.20E-05 |
| Proteobacteria | Deltaproteobacteria | Spirobacillales |  |  |  | 10.99606 | -7.00284 | 1.21E-05 |
| Bacteroidetes | [Saprospirae] | [Saprospirales] | Chitinophagaceae |  |  | 11.30409 | -7.04242 | 0.000971 |
| Verrucomicrobia | [Pedosphaerae] | [Pedosphaerales] |  |  |  | 11.66582 | -7.08802 | 1.88E-07 |
| Chlamydiae | Chlamydiia | Chlamydiales | Criblamydiaceae |  |  | 12.83613 | -7.22613 | 0.000768 |
| Chloroflexi | Ktedonobacteria | Ktedonobacterales | Ktedonobacteraceae |  |  | 12.92877 | -7.23683 | 8.32E-05 |
| TM7 |  |  |  |  |  | 13.31992 | -7.27892 | 7.41E-06 |
| Chloroflexi | Ktedonobacteria | Ktedonobacterales | Ktedonobacteraceae |  |  | 13.46318 | -7.29503 | 4.80E-06 |
| Bacteroidetes | [Saprospirae] | [Saprospirales] | Chitinophagaceae |  |  | 13.69198 | -7.32043 | 1.64E-10 |
| Bacteroidetes | Sphingobacteriia | Sphingobacteriales | Sphingobacteriaceae |  |  | 13.81973 | -7.33251 | 7.72E-05 |
| Verrucomicrobia | [Spartobacteria] | [Chthoniobacterales] | [Chthoniobacteraceae] | Unclassified | Unclassified | 14.01152 | -7.35258 | 7.94E-06 |
| Verrucomicrobia | [Spartobacteria] | [Chthoniobacterales] | [Chthoniobacteraceae] | DA101 |  | 14.06817 | -7.35813 | 0.000595 |
| TM7 | TM7-1 |  |  |  |  | 15.7731 | -7.52356 | 0.000457 |
| Bacteroidetes | Sphingobacteriia | Sphingobacteriales | Sphingobacteriaceae |  |  | 19.47777 | -7.53617 | 2.40E-08 |
| Proteobacteria | Alphaproteobacteria | Rhizobiales | Methylocystaceae |  |  | 16.37508 | -7.57769 | 0.000367 |
| Acidobacteria | Acidobacteriia | Acidobacteriales | Koribacteraceae | Candidatus Koribacter | | 17.30091 | -7.65685 | 1.89E-08 |
| Bacteroidetes | Sphingobacteriia | Sphingobacteriales | Sphingobacteriaceae |  |  | 17.33357 | -7.6594 | 5.71E-05 |
| Proteobacteria | Gammaproteobacteria | Legionellales | Coxiellaceae |  |  | 17.42658 | -7.66689 | 6.37E-12 |
| Planctomycetes | Planctomycetia | Gemmatales | Gemmataceae | Gemmata |  | 17.58605 | -7.68028 | 1.96E-08 |
| Verrucomicrobia | [Spartobacteria] | [Chthoniobacterales] | [Chthoniobacteraceae] | heteroC45_4W |  | 17.76351 | -7.69502 | 0.000337 |
| Actinobacteria | Thermoleophilia | Solirubrobacterales | Conexibacteraceae |  |  | 17.77035 | -7.69559 | 1.87E-08 |
| Proteobacteria | Gammaproteobacteria | Legionellales | Coxiellaceae |  |  | 18.65513 | -7.766 | 1.98E-08 |
| Chlamydiae | Chlamydiia | Chlamydiales | Parachlamydiaceae | Candidatus Protochlamydia | | 18.69925 | -7.76862 | 0.000336 |
| Bacteroidetes | Sphingobacteriia | Sphingobacteriales | Sphingobacteriaceae |  |  | 18.9316 | -7.78654 | 1.41E-06 |
| Proteobacteria | Gammaproteobacteria | Pseudomonadales | Pseudomonadaceae | Pseudomonas | umsongensis | 19.68947 | -7.84336 | 1.10E-06 |
| Verrucomicrobia | Verrucomicrobiae | Verrucomicrobiales | Verrucomicrobiaceae |  |  | 19.80326 | -7.85129 | 2.92E-05 |
| Proteobacteria | Alphaproteobacteria | Rhizobiales |  |  |  | 20.06836 | -7.87069 | 5.17E-13 |
| TM7 | TM7-1 |  |  |  |  | 20.20914 | -7.88089 | 6.73E-09 |
| Proteobacteria | Gammaproteobacteria | Legionellales | Legionellaceae | Unclassified | Unclassified | 20.9717 | -7.93417 | 0.000262 |
| Proteobacteria | Deltaproteobacteria | Bdellovibrionales | Bdellovibrionaceae | Bdellovibrio |  | 21.64268 | -7.97984 | 8.41E-07 |
| Bacteroidetes | Sphingobacteriia | Sphingobacteriales | Sphingobacteriaceae |  |  | 21.67243 | -7.98224 | 7.92E-13 |
| Actinobacteria | Thermoleophilia | Solirubrobacterales | |  |  | 32.60816 | -7.98979 | 5.15E-14 |
| Bacteroidetes | Sphingobacteriia | Sphingobacteriales | Sphingobacteriaceae |  |  | 22.61539 | -8.04336 | 3.27E-09 |
| Verrucomicrobia | [Spartobacteria] | [Chthoniobacterales] | [Chthoniobacteraceae] | Candidatus Xiphinematobacter | | 22.95546 | -8.06471 | 1.85E-13 |
| Proteobacteria | Gammaproteobacteria | Legionellales | Coxiellaceae |  |  | 23.58609 | -8.10393 | 9.14E-12 |
| Bacteroidetes | Sphingobacteriia | Sphingobacteriales | Sphingobacteriaceae |  |  | 24.25733 | -8.14476 | 1.78E-08 |
| Bacteroidetes | [Saprospirae] | [Saprospirales] | Chitinophagaceae |  |  | 25.04124 | -8.19013 | 7.04E-07 |
| Chlorobi |  |  |  |  |  | 25.08398 | -8.19246 | 2.61E-12 |
| Proteobacteria | Gammaproteobacteria | Xanthomonadales | Sinobacteraceae |  |  | 25.12815 | -8.19527 | 1.92E-09 |
| Proteobacteria | Betaproteobacteria | Burkholderiales | Burkholderiaceae | Salinispora | tropica | 25.70976 | -8.22823 | 3.47E-11 |
| Planctomycetes | Phycisphaerae | WD2101 |  |  |  | 26.13612 | -8.25176 | 5.85E-14 |
| TM7 | TM7-3 | EW055 |  |  |  | 27.26866 | -8.31308 | 1.35E-05 |
| Verrucomicrobia | [Spartobacteria] | [Chthoniobacterales] | [Chthoniobacteraceae] | Candidatus Xiphinematobacter | | 28.38485 | -8.37063 | 2.40E-09 |
| Proteobacteria | Alphaproteobacteria | Rhodospirillales | Acetobacteraceae |  |  | 31.11526 | -8.50349 | 7.69E-06 |
| TM6 | SJA-4 |  |  |  |  | 32.93344 | -8.58548 | 1.53E-07 |
| Proteobacteria | Alphaproteobacteria | Rhodospirillales | Rhodospirillaceae |  |  | 33.76551 | -8.62127 | 1.24E-15 |
| Proteobacteria | Betaproteobacteria | Burkholderiales | Comamonadaceae | Unclassified | Unclassified | 33.93412 | -8.62828 | 5.52E-10 |
| Acidobacteria | Acidobacteriia | Acidobacteriales | Acidobacteriaceae |  |  | 36.00608 | -8.71404 | 8.51E-08 |
| Verrucomicrobia | [Spartobacteria] | [Chthoniobacterales] | [Chthoniobacteraceae] | heteroC45_4W |  | 37.23414 | -8.76239 | 5.30E-10 |
| Firmicutes | Bacilli | Bacillales | Paenibacillaceae | Paenibacillus |  | 42.48203 | -8.95301 | 1.14E-15 |
| Verrucomicrobia | [Spartobacteria] | [Chthoniobacterales] | [Chthoniobacteraceae] | heteroC45_4W |  | 45.65415 | -9.05665 | 2.41E-16 |
| Armatimonadetes | Armatimonadia | FW68 |  |  |  | 45.73007 | -9.0592 | 2.10E-14 |
| Proteobacteria | Alphaproteobacteria | Sphingomonadales | Sphingomonadaceae | Sphingomonas | wittichii | 45.85908 | -9.063 | 1.40E-15 |
| Bacteroidetes | Sphingobacteriia | Sphingobacteriales | Sphingobacteriaceae |  |  | 46.03029 | -9.06826 | 3.86E-08 |
| Proteobacteria | Alphaproteobacteria | Unclassified | Unclassified | Unclassified | Unclassified | 47.22961 | -9.10533 | 7.27E-11 |
| Proteobacteria | Gammaproteobacteria | Legionellales | Legionellaceae | Unclassified | Unclassified | 47.82696 | -9.12351 | 4.08E-17 |
| Chlamydiae | Chlamydiia | Chlamydiales | Parachlamydiaceae |  |  | 48.51158 | -9.14411 | 3.50E-11 |
| Proteobacteria | Alphaproteobacteria | BD7-3 |  |  |  | 51.71576 | -9.23627 | 7.23E-16 |
| Proteobacteria | Betaproteobacteria | Unclassified | Unclassified | Unclassified | Unclassified | 55.54081 | -9.33938 | 9.03E-18 |
| OD1 | ZB2 |  |  |  |  | 59.59499 | -9.4408 | 1.91E-08 |
| Bacteroidetes | Sphingobacteriia | Sphingobacteriales | |  |  | 59.68989 | -9.44313 | 5.79E-08 |
| Verrucomicrobia | [Pedosphaerae] | [Pedosphaerales] | auto67_4W |  |  | 64.90125 | -9.56384 | 3.22E-18 |
| Firmicutes | Bacilli | Bacillales | Sporolactobacillaceae | Pullulanibacillus |  | 70.15414 | -9.67621 | 6.57E-09 |
| Proteobacteria | Gammaproteobacteria | Enterobacteriales | Enterobacteriaceae | Unclassified | Unclassified | 72.06969 | -9.71514 | 1.16E-16 |
| Bacteroidetes | Cytophagia | Cytophagales | Cytophagaceae | Cytophaga |  | 76.95146 | -9.80958 | 3.70E-19 |
| Planctomycetes | Planctomycetia | Gemmatales | Isosphaeraceae |  |  | 78.40645 | -9.83671 | 3.16E-09 |
| Bacteroidetes | [Saprospirae] | [Saprospirales] | Chitinophagaceae |  |  | 79.61882 | -9.8587 | 8.24E-20 |
| TM7 | TM7-3 | Unclassified | Unclassified | Unclassified | Unclassified | 87.23966 | -9.9907 | 1.08E-18 |
| Verrucomicrobia | [Spartobacteria] | [Chthoniobacterales] | [Chthoniobacteraceae] | DA101 |  | 87.98455 | -10.003 | 1.30E-20 |
| Verrucomicrobia | [Spartobacteria] | [Chthoniobacterales] | [Chthoniobacteraceae] | Chthoniobacter |  | 88.23041 | -10.007 | 2.63E-09 |
| Proteobacteria | Alphaproteobacteria | Caulobacterales | Caulobacteraceae | Caulobacter |  | 96.56647 | -10.1373 | 1.95E-20 |
| Proteobacteria | Gammaproteobacteria | Enterobacteriales | Enterobacteriaceae | Serratia |  | 116.2887 | -10.4054 | 6.80E-21 |
| Planctomycetes | Phycisphaerae | WD2101 |  |  |  | 148.6224 | -10.7592 | 3.56E-23 |
| Verrucomicrobia | [Pedosphaerae] | [Pedosphaerales] | auto67_4W |  |  | 166.3218 | -10.9216 | 5.22E-24 |
| Bacteroidetes | Sphingobacteriia | Sphingobacteriales | |  |  | 10.09099 | -22.2814 | 3.17E-14 |

Criteria for inclusion: BaseMean > 5 and padj < 0.0001. Brackets indicate putative taxonomy based upon phylogenetic placement as given in the Greengenes taxonomy. BaseMean is the mean of normalized counts for all samples; padj is the Benjamini–Hochberg adjusted *P* value.

Supplementary Table S5. Keystone taxa of topology properties of tomato rhizosphere that treated by upland and forest MF microbiome network

Supplementary Table S6. Topology properties of tomato rhizosphere that treated by alluvial, paddy, forest and upland MF microbiome network

| Network properties | Alluvial | Paddy | Forest | Upland |
| --- | --- | --- | --- | --- |
| Total nodes ^a^ | 126 | 143 | 140 | 174 |
| Total edges ^b^ | 968 | 409 | 281 | 765 |
| Average Degree ^c^ | 7.683 | 2.86 | 2.007 | 4.397 |
| Network Diameter ^d^ | 7 | 6 | 5 | 7 |
| Network Density ^e^ | 0.061 | 0.020 | 0.014 | 0.025 |
| Average Clustering Coefficient ^f^ | 0.193 | 0.137 | 0.158 | 0.176 |
| Average Path Length ^g^ | 2.34 | 2.127 | 1.871 | 2.647 |
| The number of module^h^ | 6 | 19 | 16 | 10 |

^a^ Microbial taxa with at least one significant correlation

^b^ Number of connections/correlations generated by MENA analysis

^c^ The average number of connections per node in the network, that is, the node connectivity (Gephi).

^d^ The longest distance between nodes in the network, measured in number of edges (Gephi).

^e^ Measure how close the network is to complete (Gephi).

^f^ How nodes are embedded in their neighborhood and the degree to which they tend to cluster together (Gephi).

^g^ Average network distance between all pair of nodes or the average length off all edges in the network (Gephi).

^h^ The presence of different groups of nodes with high number of edges (correlations) within and with some degree of independencies between groups
